# Large language model performance versus human expert ratings in automated suicide risk assessment

**DOI:** 10.1038/s41598-025-22402-7

**Published:** 2025-11-10

**Authors:** Julia Thomas, Zohar Elyoseph, Lars Kuchinke, Gunther Meinlschmidt

**Affiliations:** 1 krisenchat gGmbH, Berlin, Germany; 2 Division of Clinical Psychology and Epidemiology, Department of Psychology, University of Basel, Basel, Switzerland; 3 Division of Clinical Psychology and Cognitive Behavioural Therapy, International Psychoanalytic University (IPU) Berlin, Berlin, Germany; 4 Department of Counseling and Human Development, Faculty of Education, University of Haifa, Haifa, Israel; 5 Imperial College London, London, UK; 6 Psychological Methods and Evaluation, International Psychoanalytic University (IPU) Berlin, Berlin, Germany; 7 Department of Clinical Psychology and Psychotherapy – Methods and Approaches, Trier University, 54286 Trier, Germany; 8 Department of Digital and Blended Psychosomatics and Psychotherapy, Psychosomatic Medicine, University Hospital and University of Basel, Basel, Switzerland; 9 Department of Psychosomatic Medicine, University Hospital and University of Basel, Basel, Switzerland

**Keywords:** Natural language processing, Retrieval augmentation, Machine learning, Psychometry, Benchmarking, Psychology, Health care

## Abstract

Large Language Models’ (LLMs) potential for psychological diagnostics requires systematic evaluation. We aimed to investigate conditions for reliable and valid psychological assessments, focusing on suicide risk evaluation in clinical data by comparing LLM-generated ratings with human expert ratings across model configurations., analyzing 100 youth crisis text line conversation transcripts rated by four experts using the Nurses’ Global Assessment of Suicide Scale (NGASR). Using Mixtral-8x7B-Instruct, we generated ratings across three temperature settings and prompting styles (zero-shot, few-shot, chain-of-thought). Across configurations we compared (a) inter-rating-reliability for AI-generated NGASR risk and sum scores, (b) LLM-to-human observer agreement regarding sum score, risk category, and item, using Krippendorff’s α, (c) classification metrics of risk categories and individual items against human ratings. LLM configuration strongly influenced assessment reliability. Zero-shot prompting at temperature 0 yielded perfect inter-rating reliability (α = 1.00, 95% CI: [1–1] for high & very high risk), while few-shot prompting showed best human-AI agreement for very high risk (α = 0.78, 95% CI: [0.67–0.89]) and strongest classification performance (balanced accuracy 0.54–0.71). Lower temperatures consistently improved reliability and accuracy. However, critical clinical items showed poor validity. Our findings establish optimal conditions (zero temperature, task-specific prompting) for LLM-based psychological assessment. However, inconsistent clinical item performance and only moderate LLM-to-human observer agreement limit LLMs to initial screening rather than detailed assessment, requiring careful parameter control and validation.

**Supplementary Information:**

The online version contains supplementary material available at 10.1038/s41598-025-22402-7.

## Introduction

Large Language Models (LLM) are neural networks that predict text sequences using conditional word probabilities^1^. Through self-supervised learning, they process language by optimizing billions of parameters for text prediction^2^. These “foundational models” execute various tasks based on textual instructions or “prompts”^3^. LLMs demonstrate emergent capabilities in processing and reasoning by identifying complex word associations and developing implicit knowledge through vast training^4–6^.

LLMs show promise in clinical psychology due to their language processing capabilities^7^. They support medical information retrieval^8^, treatment decisions^9^, clinical summarization^10^, and patient education^11^. Their extensive pre-training on large-scale text corpora containing medical and psychological literature enables LLMs to draw upon patterns from medical and psychological knowledge encountered during training^11^. AI-powered applications offer 24/7 availability, streamlined processing through automated assessment and rapid analysis of large volumes of clinical text^12^, and reduced administrative burden^3^, while requiring appropriate human oversight for clinical decision-making. LLMs’ contextual embeddings capture language nuances^13,14^and individual usage patterns^15^, while showing evidence of cultural sensitivity in suicide risk assessments across different demographic groups^16^.

Although not explicitly designed for psychological assessments, LLMs can be adapted through techniques like structured evaluation-based scoring^17^. However, ensuring high-quality benchmarks for clinical decision-making remains challenging due to “hallucinations”—generating plausible but incorrect outputs^18^. This phenomenon, stemming from LLMs’ probabilistic nature and lack of intrinsic truth understanding^19^, poses a significant obstacle to achieving clinical-grade accuracy and reliability in LLM-based assessments^20^. This holds especially true in high risk domains of psychology and medicine.

One of these domains is suicide prevention. Suicide remains a leading global mortality cause^21^, presenting an urgent need for cost-effective tools for prevention and monitoring^22–24^. Crisis text lines have demonstrated potential in suicide prevention by offering accessible support, making them an ideal testing ground for AI applications. The integration of LLM systems into mental health care holds particular promise for suicide prevention, where timely interventions can save lives.

Recent studies demonstrate LLMs’ potential in psychiatric risk assessment. GPT-4 matched mental health professionals’ assessment capabilities^16,25^, showed enhanced risk factor detection^26^, and analyzed suicide-related media content effectively^27^. GPT-4 also achieved 0.6 precision in suicide plan prediction versus clinicians’ 0.7, with higher sensitivity (0.62 vs 0.53;^28^). LLM analysis of crisis hotlines achieved 76% F1 score, outperforming manual assessments and traditional deep learning^29^. Levkovich and Omar^30^ systematically reviewed BERT-based and large language models (including GPT, Llama, and BERT derivatives) across 29 studies, finding that these models frequently outperformed mental health professionals in early detection and prediction capabilities. However, their review highlighted the need for ethical considerations and expert collaboration, emphasizing that superior technical performance does not eliminate the requirement for clinical oversight. Moreover, explicit validation studies with clinical data remain limited.

While these studies demonstrate promising potential, critical gaps remain in understanding how to achieve reliable and valid LLM-based clinical assessments. First, existing research hasn’t systematically examined how different LLM configurations affect assessment reliability and validity. Second, while various prompting strategies exist, their comparative effectiveness for clinical assessment remains untested. Third, the impact of temperature settings on clinical judgment reliability is unexplored, particularly for high-stakes decisions. Finally, no studies have conducted item-level analyses to identify which clinical assessment components are most suitable for LLM evaluation. To address these gaps, we investigated LLM agents with retrieval-augmented generation (RAG) for structured psychological suicide assessments by measuring agreement between human and LLM ratings. We examined (1) the impact of various prompting styles (zero-shot, few-shot, and chain-of-thought) on reliability and validity, (2) observer agreement and classification performance across different operational settings to human expert raters, and (3) conducted granular analysis of item-specific metrics to assess which individual items were most amenable to automated assessment.

## Methods

### Study design

**Fig. 1:**
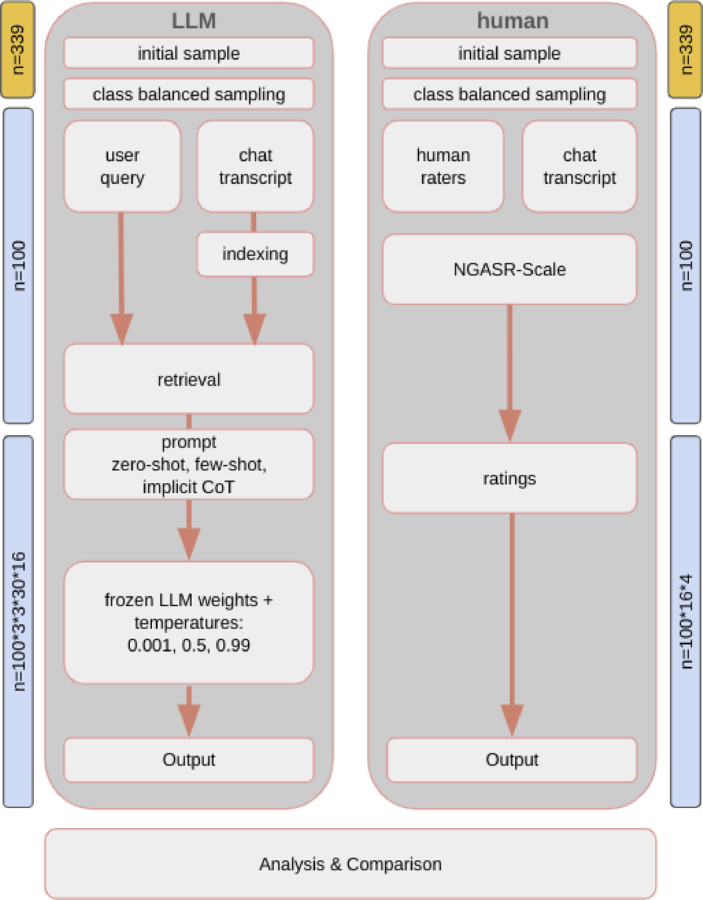
Methodological framework: data processing pipeline for human-LLM^a^ comparison in NGASR^b^ suicide risk assessment, assessing the presence of suicide risk factors based on n = 100 session transcripts of german youth crisis helpline users between 2021-11-30 and 2022-04-30. ^a^ LLM, large language model. ^b^ NGASR, Nurses’ Global Assessment of Suicide Risk

We present the study design in Fig. 1. The study analyzed chat transcripts from the German crisis text line, krisenchat (Fig. 1). Four expert raters independently scored 16 items of the NGASR scale (Cutcliffe & Barker, 2004) to assess suicide risk. An LLM agent generated similar ratings using varied temperature values and prompting styles. We compared human and AI evaluations through interrater reliability, observer agreement, and classification metrics across operational settings.

### Data preparation

This study analyzed chat transcripts from krisenchat, a German preclinical crisis intervention service for individuals up to 25 years old^31^. The data comprised counseling sessions conducted between 2021-11-30, and 2022-04-30, with transcripts representing complete counseling histories.

#### Sample selection and stratification

From an initial pool of n = 439 labeled cases, we selected n = 100 cases using stratified random sampling to ensure balanced representation across NGASR-assessed risk levels. This sample size was determined by computational constraints, as each case required extensive processing (4320 LLM ratings per transcript across 16 items × 3 temperature settings × 3 prompting styles × 30 repetitions). Processing all 439 cases would have exceeded our available computational resources, while stratified random sampling with balanced representation helped mitigate potential selection bias. The sample was equally distributed with n = 25 cases in each risk category: low (< 4), moderate (5–8), high (9–12), and very high (> 12). Study participation was restricted to female participants with a minimum age of 14 years who were seeking help for themselves, excluding cases of help-seeking for others as well as male and diverse gender cases.

The restriction to female participants was implemented for three reasons: female users comprised > 85% of crisis line contacts during the study period; research indicates significant gender differences in suicidal behavior patterns and communication styles during adolescence^32^, suggesting mixed-gender analysis could introduce confounding variables; and the limited sample size necessitated a homogeneous population to ensure adequate statistical power for reliable LLM assessment.

We used stratified sampling to achieve equal base rates (25% each) across four NGASR risk levels, differing from natural crisis population prevalence to ensure adequate statistical power across all categories.

#### Data processing

To maintain internal validity, transcripts were preserved without modifications. Risk levels were determined using majority-voted NGASR sum scores from four independent clinical experts. For binary classifications (presence/absence of risk factors), a threshold of greater than 50% agreement among human raters or LLM ratings was used to establish positive items, 50:50 situation would result in a negative item.

### Measures

The NGASR scale, developed by Cutcliffe and Baker^33^ and translated into German by Kozel et al.^34,35^, is a structured 16-item questionnaire assessing evidence-based suicide risk factors, not individual suicide probability. The scale encompasses a range of risk factors: hopelessness, recent stress events, hallucinations/delusions, depression, social withdrawal, suicidal intention, suicide plans, family psychiatric/suicide history, recent losses, psychotic disorder, widowhood, previous attempts, poor socioeconomic conditions, substance abuse, terminal illness, and multiple hospitalizations. Five items—hopelessness, depression, suicidal plans, recent losses, and previous attempts—carry triple weight in scoring due to their elevated predictive value. Total scores indicate risk levels categorized as low (4 or below), moderate (5–8), high (9–11), and very high (12 and above). The German validation study demonstrated strong psychometric properties, with median item-wise observer agreements of 0.64 in Cohen’s Kappa (K) and 0.85 in Gwet’s AC1(AC1), while sum score agreements reached 0.90 and 0.91 in absolute agreement of Intra-Class-Correlation (ICC) and consistency, respectively.

#### Rating procedure

Our inter-rater approach with four independent experts, combined with regression bias correction, explicitly acknowledges that clinical assessment research consistently shows rater variability often exceeds candidate variability^36^. This statistical approach accounts for inherent uncertainties in human clinical decision-making while providing the most robust available approximation of clinical consensus. Four independent expert raters from a specialized suicide and self-harm counseling unit^37^ conducted the clinical assessments. All raters were trained clinical psychologists with formal academic qualifications in psychology (minimum bachelor’s degree, with the majority holding master’s degrees or currently enrolled in postgraduate psychotherapy training programs). Each rater was employed as a counselor within the crisis service’s suicide prevention unit, providing them with specialized experience in suicide risk assessment and crisis intervention with youth populations.The raters underwent training on NGASR items through panel ratings and group discussions using non-study cases prior to conducting assessments. Each rater independently evaluated the complete set of counseling transcripts across all NGASR items. Inter-rater agreement was evaluated using Krippendorff’s α^38,39^. To maintain consistency and prevent observer drift, integrity discussions were conducted between rating sessions^40^, allowing raters to share insights and standardize their approach without modifying existing ratings. For analysis purposes, individual ratings were aggregated using majority voting, where agreement from more than 50% of raters established positive cases. Final sum scores and risk level assignments were calculated based on these aggregated ratings, incorporating the differential item weights specified in the NGASR manual.

### LLM framework and implementation

We implemented a framework using Mixtral-8x7B, which employs Sparse Mixture of Experts (SMoE) architecture. Unlike traditional models that use all parameters for every input, SMoE selectively activates only a subset of specialized ‘expert’ networks within the model based on the specific content being processed^41^. This targeted activation allows for more focused and specialized processing of clinical text, as relevant expert networks can concentrate on domain-specific patterns^42^. The second embeddings model converts conversations into numerical embeddings using an instructor-transformer model based on T5 architecture^43^, enabling similarity comparisons via euclidean distance^20,44,45^. Our RAG approach anchors LLM responses to conversation context^42^, by dynamically injecting relevant conversation context to reduce hallucinations and improve response accuracy^46^.

Implementation parameters included: 500-token chunks with 25% overlap, top 5 conversation chunks, and 0.95 similarity threshold. We tested temperature settings of 0.0, 0.5, and 1.0 to control output randomness, with lower values producing more deterministic results^47,48^. Temperature settings above 1.0 were not explored based on literature recommendations for accuracy-critical tasks^49 ^and empirical evidence showing substantial accuracy degradation at temperatures above 1.25, with studies documenting up to 51% accuracy loss at temperature 1.75 and increased incoherent outputs^51^.

**Fig. 2:**
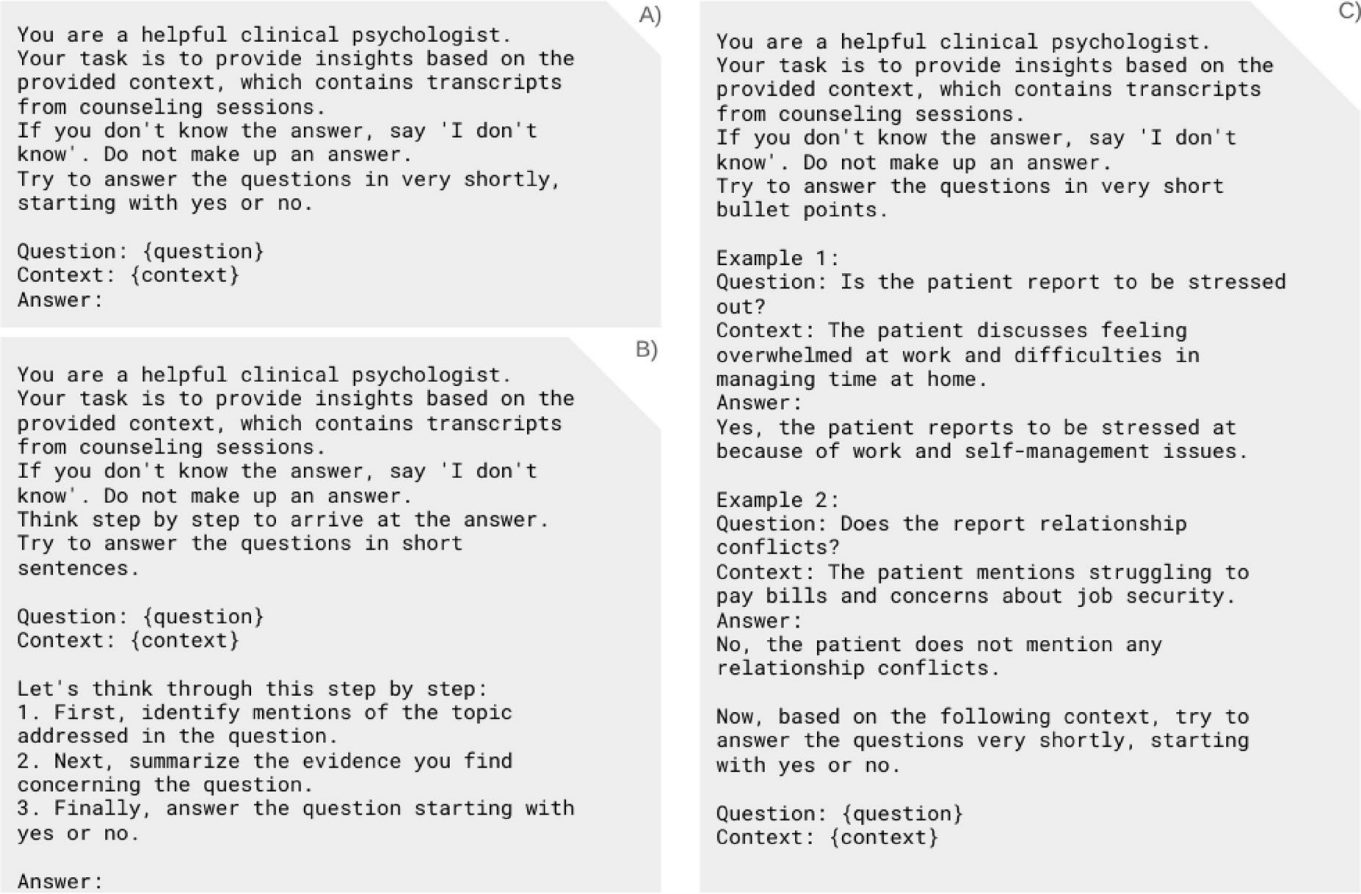
Vignettes of different prompting styles for LLM^a^-based item-assessment: (**A**) zero-shot, (**B**) chain-of-thought, and (**C**) few-shot prompting examples, showing template structure and example interactions for each approach (N = 3 prompting styles), assessing the presence of suicide risk factors based on n = 100 session transcripts of german youth crisis helpline users between 2021-11-30 and 2022-04-30. ^a^LLM, large language model.

The study explored three distinct prompting styles: zero-shot, few-shot, and chain-of-thought. Zero-shot prompting presented questions directly from the scale manual without examples, relying on the model’s pre-existing knowledge to interpret and rate counseling transcripts. Few-shot prompting enhanced contextual understanding by providing carefully selected positive and negative examples prior to the rating task, while avoiding potential answer bias through example selection^50^. Chain-of-thought (CoT) prompting encouraged structured clinical reasoning by requiring step-by-step articulation of the assessment process, enabling insight into the model’s decision-making approach. Refer to (Fig. 2) for exemplary prompting style formulations.

Each prompting style incorporated a RAG context, role specification, and clear output requirements. The implemented framework can be represented as:

$$Zero - Shot - Prompt \, = \, RAG_{context} + \, P_{role} + \, P_{output \, specification} + \, P_{question}$$

$$Few - Shot - Prompt = \, RAG_{context} + \, P_{role} + \, P_{output \, specification} + \, P_{examples} + \, P_{question}$$

$$Chain - of - Thought - Prompt \, = \, RAG_{context} + \, P_{role} + \, P_{output \, specification} + \, P_{question} + \, P_{COT - Instruction}$$

The LLM generated 4320 ratings per transcript (16 NGASR items × 3 temperature settings × 3 prompting styles × 30 repetitions). We termed each prompting style and temperature combination an *operational configuration*. For each configuration, we aggregated individual item ratings through majority voting, requiring > 50% agreement to establish positive cases. We then calculated risk levels and sum scores following the NGASR manual’s scoring rules.

### Statistical analysis

#### Descriptive analysis

For descriptive analysis, we characterized sociodemographic characteristics (age, gender) and service usage behaviors (number of counselor messages, number of chatter messages, number of counseling sessions) using frequencies for categorical variables, mean, median, range and standard deviation for normally distributed continuous variables, as detailed in Table 1. These statistics were stratified by risk level, with differences between risk levels evaluated using Chi-square tests for categorical variables and Kruskal–Wallis-Tests for continuous variables violating the normal distribution.

**Table 1:** Demographic and clinical characteristics of crisis helpline users stratified by suicide risk level (N = 100), assessing the presence of suicide risk factors based on n = 100 session transcripts of german youth crisis helpline users between 2021-11-30 and 2022-04-30.

NGASR^a^ risk level	Age in years Mean ± SD Median (Min–Max)	NGASR^a^ sum score Mean ± SD Median (Min–Max)	Number of counselor messages Mean ± SD Median (Min–Max)	Number of chatter messages Mean ± SD Median (Min–Max)	Number of counseling sessions Mean ± SD Median (Min–Max)
Low	16.8 ± 2.66 17.0 (14–24)	2.12 ± 1.59 2.0 (0–4)	143.32 ± 208.69 67.0 (9–763)	206.52 ± 414.45 74.0 (7–2026)	16.00 ± 26.01 7.0 (1–117)
Moderate	16.0 ± 2.25 15.0 (14–23)	6.32 ± 1.18 6.0 (5–8)	403.96 ± 769.46 110.0 (17–3666)	512.00 ± 1071.12 158.0 (14–5221)	40.00 ± 75.60 13.0 (1–361)
High	16.76 ± 2.76 16.0 (14–25)	10.20 ± 0.87 10.0 (9–11)	487.88 ± 770.58 275.0 (41–3712)	590.68 ± 989.85 294.0 (48–4890)	45.80 ± 68.13 21.0 (2–326)
Very high	17.32 ± 2.72 17.0 (14–24)	14.56 ± 2.40 14.0 (12–21)	571.20 ± 624.93 303.0 (19–2035)	643.48 ± 723.57 370.0 (24–2629)	49.44 ± 57.75 33.0 (2–217)

Values presented as Mean ± Standard Deviation (SD) with median and min–max for age, NGASR^a^ sum scores, message counts, and session counts.

^a^ NGASR, Nurses’ Global Assessment of Suicide Risk.

#### Reliability analysis

Reliability is assessed as internal consistency among LLM ratings (α coefficients for LLM-to-LLM agreement). We assessed LLM measurement reliability of NGASR risk levels using Krippendorff’s α coefficients, treating each binary risk level as an independent rater. Krippendorff’s α accommodates multiple scale types (binary, ordinal, metric), enabling consistent comparison across NGASR items, risk levels, and sum scores. We used established agreement thresholds: perfect (1), substantial (≥ 0.80), moderate (0.67–0.79), weak (0.60–0.66), and poor (< 0.60). Negative values indicated systematic disagreement. Uncertainty was quantified through bootstrapping (1000 resamples) to compute 95% confidence intervals for α values per operational configuration.

#### Observer agreement analysis

Observer agreement was evaluated as external validity by measuring LLM-to-human concordance using regression bias-corrected Krippendorff’s α coefficients. Using individual item ratings, we calculated sum scores and risk levels for each LLM and human rating. To evaluate validity against human ratings, we employed Krippendorff’s α coefficient with regression bias correction, accounting for nested rater groups of human raters and LLM ratings. This correction adjusts for the fact that overall agreement between groups is limited by within-group agreement levels, providing more accurate estimates of true inter-group agreement. Separate α coefficients were computed across risk levels, and sum scores aggregated per operational configuration. The overall α value was corrected for within-group agreement using:

$$\alpha \_{\text{corrected}}\, = \,\alpha \_observed\, + \,\beta \left( {\alpha \_expected{-\!\!-}\alpha \_observed} \right)$$

where α_corrected represents the regression bias corrected Krippendorff’s α, α_observed is the originally calculated α, α_expected is the expected α value under the null hypothesis (typically 0), and β represents the regression coefficient capturing the relationship between within-group and between-group agreement rates. This coefficient essentially determines how much the observed agreement should be adjusted based on within-group rating patterns. We quantified uncertainty through bootstrapping (1000 resamples) to compute 95% confidence intervals for individual α values of risk levels per operational configuration.

#### Classification performance analysis

We derived final ratings through majority voting (> 50% agreement) from LLM outputs (30 ratings per item/prompt/temperature combination) and human ratings (4 per item). NGASR sum scores were calculated and categorized into four risk levels. As per each risk level, we computed binary classification metrics by comparing that level against all others combined (e.g., “high risk” vs. “not high risk”).

We assessed validity against the human gold standard through balanced accuracy, sensitivity, and specificity. Balanced accuracy addresses imbalanced sample rates by measuring detection ability for both present and absent risk factors, crucial for rare but critical symptoms. To account for potential base rate effects, we employed balanced accuracy as our primary metric, as this measure averages sensitivity and specificity to provide an unbiased performance assessment regardless of class distribution. Balanced accuracy is essential in suicide risk assessment, where standard accuracy can misleadingly appear high due to rare outcomes of interest while masking poor sensitivity to said rare outcomes. Sensitivity measures ability to detect present risk factors relative to positive case base rate, critical for identifying potential dangers. Specificity evaluates correct identification of true negatives compared to negative case base rate, important for avoiding false alarms and incurring costly and unnecessary treatment.

Performance above respective base rates indicates meaningful discriminative ability, distinguishing true predictive performance from class distribution effects. We calculated 95% confidence intervals through bootstrapping (1000 resamples) for each operational configuration. Values exceeding 0.5 demonstrate above-random performance.

#### Item specific analysis

To evaluate automation potential across risk factors, we conducted item-specific analyses using deterministic model outputs (temperature 0) from different prompting approaches. Item observer agreement per prompting style was evaluated through Krippendorff’s α coefficient with regression bias correction.

Final item classifications were derived via majority voting for each prompting style and compared against human consensus ratings. We evaluated classification performance through balanced accuracy, sensitivity, and specificity metrics, comparing these against respective base rates to determine significant improvements over chance-level performance.

#### Error analysis

We analyzed cases where LLM ratings diverged from expected clinical reasoning through qualitative assessment. Our examination of chain-of-thought outputs revealed patterns in failed assessments. We analyzed both content and structure of the model’s clinical reasoning process, focusing on deviations from standard clinical judgment.

### Tools and software

Analyses were conducted using Python 3.8 on a Google Cloud Platform Kubernetes cluster. A 5-bit quantized Mixtral-8x7B model was deployed on a 24 GB L4 GPU machine using Ollama. The workflow utilized LangChain^52^ for LLM interaction and retrieval augmentation, Pandas^53^ for data manipulation, Pingouin^54^ and Krippendorff^55^ packages for statistical calculations, and Seaborn^56^ and Matplotlib^57^ for visualizations and re for regular expression string matching^58^.

### Ethical considerations

All methods in this study were carried out in accordance with relevant guidelines and regulations. All experimental protocols were approved by the Ethics Committee of the International Psychoanalytic University (IPU) Berlin (approval number: 2023_08). Informed consent was obtained from all subjects through krisenchat’s terms of service, which explicitly state that user data may be used for research purposes without direct identification of individuals. All personally identifiable information was removed from chat transcripts during preprocessing. The study utilized existing data from the crisis helpline, and participants were not compensated as this was a secondary analysis of routine service data. Research was performed in accordance with the Declaration of Helsinki.

### Research standards

This study followed CONSORT-AI and TRIPOD-AI reporting guidelines (Tables S4 and S5).

## Results

### Descriptive analysis

The analysis included 100 cases stratified by NGASR-assigned risk levels: low (< 4), moderate (5–8), high (9–12), and very high (> 12), randomly sampled from 439 labeled cases.

Chi-square tests indicated group differences in age, with very high risk cases showing higher overall age. Refer to (Table 1) for more detail. Analysis of demographic and interaction variables across risk levels revealed no significant differences. However, messaging behaviors differed significantly by risk level. ANOVA assumptions were violated for all messaging variables due to non-normal distributions (Shapiro–Wilk tests, all *p* < 0.001), requiring non-parametric analysis. Kruskal–Wallis tests revealed significant differences in counselor message counts (H(3) = 19.418, *p* < 0.001), user message counts (H(3) = 18.708, *p* < 0.001), and session counts (H(3) = 14.413, *p* = 0.002). Median values showed clear escalation with risk level: counselor messages increased from 67 (low risk) to 303 (very high risk), user messages from 74 to 370, and sessions from 7 to 33, indicating more service engagement among higher-risk individuals.

### Reliability analysis

Human raters demonstrated varying reliability across risk levels, from high reliability in low risk cases (α = 0.91 [0.85, 0.97]) to weaker agreement in high risk assessments (α = 0.63 [0.51, 0.74]). LLM reliability analysis revealed distinct patterns across risk levels and prompting approaches. For low risk cases, few-shot prompting at temperature 0 achieved numerically highest reliability (α = 0.98 [0.95, 1.02]), though confidence intervals overlap with human reliability (α = 0.91 [0.85, 0.97]), hence not providing support for differences in performance. Zero-shot demonstrated perfect reliability (α = 1.00) for high and very high risk levels, reflecting the deterministic nature of temperature 0 settings. While numerically higher than human agreement (α = 0.63 [0.51, 0.74] for high risk and α = 0.76 [0.66, 0.87] for very high risk), the clinical significance of perfect deterministic agreement warrants careful interpretation. Please refer to (Table 2) for a detailed lineout of human observer agreement.

**Table 2:** Human inter-rater reliability analysis across items and risk levels (N = 400 ratings), assessing the presence of suicide risk factors based on n = 100 session transcripts of german youth crisis helpline users between 2021-11-30 and 2022-04-30.

Metric type	Low risk	Moderate risk	High risk	Very high risk	Sum score
Human reliability	0.91 95% CI: [0.85, 0.97]	0.67 95% CI: [0.57, 0.78]	0.63 95% CI: [0.51, 0.74]	0.76 95% CI: [0.66, 0.87]	−0.02 95% CI: [−0.16, 0.11]

Values represent Krippendorff’s α coefficients shown as Mean with 95% Confidence Intervals. Analysis based on ratings from 4 independent clinical experts. Perfect agreement cases coded as 1.0. Negative values indicate systematic disagreement.

**Fig. 3:**
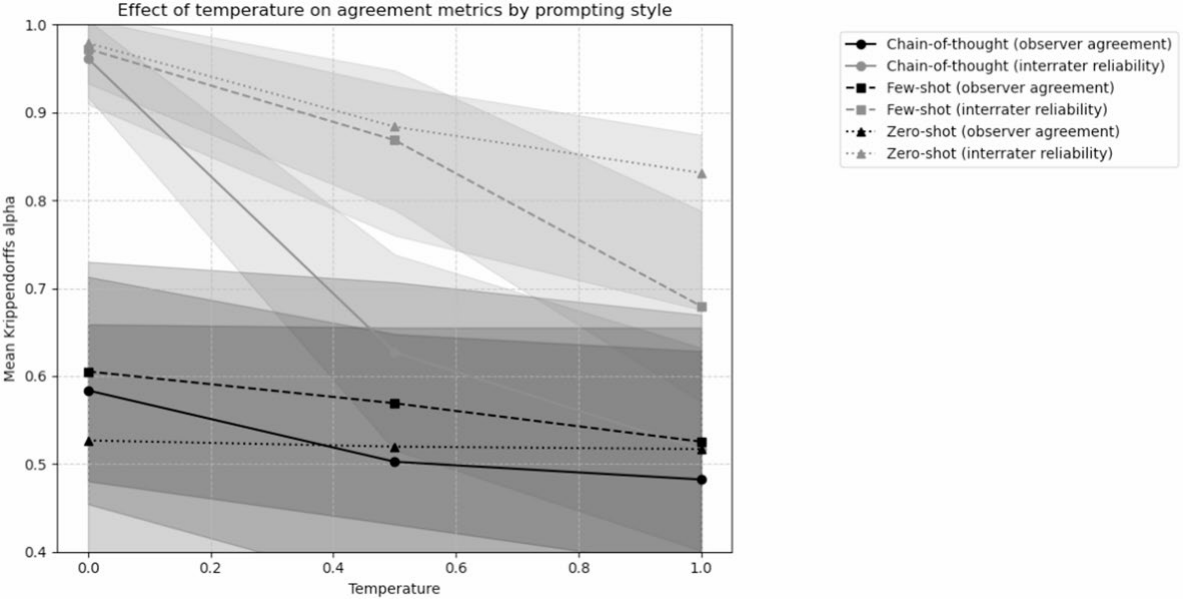
Comparative analysis of LLM^a^ inter-rating reliability and human-to-LLM^a^ observer agreement across Temperature Settings (0, 0.5, 1) and Prompting Styles (zero-shot, few-shot, chain-of-thought), measured using Krippendorff’s α and regression bias corrected Krippendorff’s α (N = 48,000 per configuration), assessing the presence of suicide risk factors based on n = 100 session transcripts of German Youth Crisis Helpline Users between 2021-11-30 and 2022-04-30. ^a^ LLM, large language model.

Temperature increase markedly affected reliability across prompting styles. Chain-of-thought showed the most pronounced degradation, with low risk reliability dropping from α = 0.97 [0.93, 1.01] to α = 0.61 [0.50, 0.72] between temperature 0 and 1 (Fig. 3). Few-shot demonstrated more stability, particularly in very high risk cases (α = 0.97 [0.92, 1.01] at temperature 0 to α = 0.80 [0.72, 0.89] at temperature 1). Sum scores showed systematic disagreement in both human (α = −0.02 [− 0.16, 0.11]) and LLM ratings, with the effect amplifying at higher temperatures. For all LLM inter-rating Reliability and Observer Agreement values refer to (Table 3).

**Table 3:** LLM^a^ inter-rating reliability and observer agreement of risk levels and sum score compared across prompting style and temperature (N = 48,000 per configuration), assessing the presence of suicide risk factors based on n = 100 session transcripts of german youth crisis helpline users between 2021-11-30 and 2022-04-30.

Risk level	Temp	Metric	Chain-of-thought	Few-shot	Zero-shot
Low	0	LLM reliability	0.97 95% CI: [0.93, 1.01]	0.98 95% CI: [0.95, 1.02]	0.96 95% CI: [0.91, 1.01]
		Observer agreement	0.72 95% CI: [0.62, 0.83]	0.78 95% CI: [0.68, 0.88]	0.39 95% CI: [0.24, 0.53]
	0.5	LLM reliability	0.75 95% CI: [0.65, 0.85]	0.93 95% CI: [0.87, 0.99]	0.80 95% CI: [0.70, 0.91]
		Observer agreement	0.69 95% CI: [0.58, 0.81]	0.78 95% CI: [0.68, 0.89]	0.42 95% CI: [0.27, 0.57]
	1	LLM reliability	0.61 95% CI: [0.50, 0.72]	0.87 95% CI: [0.80, 0.95]	0.77 95% CI: [0.66, 0.87]
		Observer agreement	0.67 95% CI: [0.54, 0.79]	0.78 95% CI: [0.67, 0.88]	0.41 95% CI: [0.26, 0.56]
Moderate	0	LLM reliability	0.98 95% CI: [0.94, 1.01]	0.97 95% CI: [0.94, 1.01]	0.96 95% CI: [0.91, 1.01]
		Observer agreement	0.33 95% CI: [0.19, 0.48]	0.33 95% CI: [0.18, 0.47]	0.30 95% CI: [0.15, 0.45]
	0.5	LLM reliability	0.40 95% CI: [0.28, 0.53]	0.83 95% CI: [0.74, 0.92]	0.78 95% CI: [0.68, 0.88]
		Observer agreement	0.23 95% CI: [0.06, 0.41]	0.31 95% CI: [0.16, 0.46]	0.25 95% CI: [0.10, 0.40]
	1	LLM reliability	0.27 95% CI: [0.15, 0.39]	0.55 95% CI: [0.42, 0.69]	0.68 95% CI: [0.56, 0.79]
		Observer agreement	0.20 95% CI: [0.03, 0.37]	0.30 95% CI: [0.13, 0.46]	0.26 95% CI: [0.10, 0.42]
High	0	LLM reliability	0.94 95% CI: [0.89, 1.00]	0.96 95% CI: [0.92, 1.01]	1.00 95% CI: [–, –]
		Observer agreement	0.55 95% CI: [0.41, 0.70]	0.53 95% CI: [0.39, 0.67]	0.67 95% CI: [0.55, 0.80]
	0.5	LLM reliability	0.58 95% CI: [0.46, 0.70]	0.80 95% CI: [0.70, 0.90]	0.95 95% CI: [0.90, 1.00]
		Observer agreement	0.34 95% CI: [0.17, 0.51]	0.47 95% CI: [0.30, 0.64]	0.66 95% CI: [0.53, 0.79]
	1	LLM reliability	0.50 95% CI: [0.38, 0.62]	0.49 95% CI: [0.35, 0.62]	0.88 95% CI: [0.80, 0.96]
		Observer agreement	0.35 95% CI: [0.17, 0.52]	0.34 95% CI: [0.15, 0.52]	0.65 95% CI: [0.52, 0.78]
Very high	0	LLM reliability	0.95 95% CI: [0.91, 1.00]	0.97 95% CI: [0.92, 1.01]	1.00 95% CI: [–, –]
		Observer agreement	0.73 95% CI: [0.61, 0.85]	0.78 95% CI: [0.67, 0.89]	0.75 95% CI: [0.64, 0.86]
	0.5	LLM reliability	0.77 95% CI: [0.68, 0.87]	0.91 95% CI: [0.85, 0.98]	1.00 95% CI: [–, –]
		Observer agreement	0.74 95% CI: [0.62, 0.86]	0.71 95% CI: [0.58, 0.83]	0.75 95% CI: [0.64, 0.86]
	1	LLM reliability	0.69 95% CI: [0.58, 0.80]	0.80 95% CI: [0.72, 0.89]	1.00 95% CI: [–, –]
		Observer agreement	0.72 95% CI: [0.60, 0.84]	0.69 95% CI: [0.57, 0.81]	0.75 95% CI: [0.64, 0.86]
Sum Score	0	LLM reliability	0.78 95% CI: [0.66, 0.89]	0.86 95% CI: [0.76, 0.95]	0.90 95% CI: [0.81, 0.98]
		Observer agreement	−0.59 95% CI: [−0.71, −0.47]	−0.58 95% CI: [−0.65, −0.50]	−0.40 95% CI: [−0.58, −0.22]
	0.5	LLM reliability	−0.18 95% CI: [−0.30, −0.06]	0.41 95% CI: [0.25, 0.56]	0.34 95% CI: [0.19, 0.50]
		Observer agreement	−0.90 95% CI: [−0.94, −0.85]	−0.76 95% CI: [−0.83, −0.69]	−0.64 95% CI: [−0.76, −0.51]
	1	LLM reliability	−0.36 95% CI: [−0.41, −0.32]	−0.12 95% CI: [−0.26, 0.01]	0.11 95% CI: [−0.04, 0.26]
		Observer agreement	−0.90 95% CI: [−0.94, −0.86]	−0.85 95% CI: [−0.94, −0.77]	−0.74 95% CI: [−0.83, −0.64]

Values shown as Mean with 95% Confidence Intervals. LLM reliability represents agreement among LLM ratings (llm_α); Observer agreement represents regression-bias corrected agreement between human and LLM ratings (corrected_α). All metrics calculated using Krippendorff’s α with perfect agreement coded as 1.0. Negative values indicate systematic disagreement. ^a^ LLM ,  large language model.

### Observer agreement analysis

**Fig. 4:**
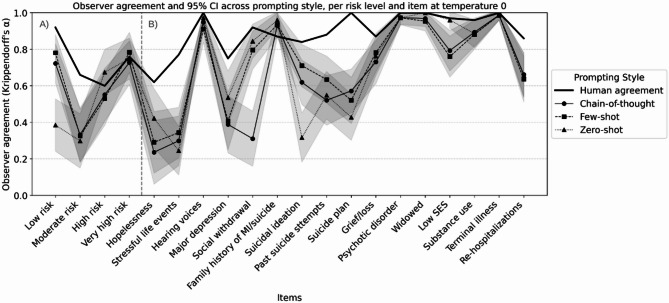
Regression bias corrected observer agreement values comparing human (n = 4) and LLM^a^ Ratings (N = 30) across prompting styles at temperature 0, panel (**A**): aggregated per Iiem, panel (**B**): aggregated per NGASR^b^ Risk Level (N = 2700 each), measured with Krippendorff’s α, values shown as Mean and 95% Confidence Interval, assessing the presence of suicide risk factors based on n = 100 session transcripts of german youth yrisis helpline users between 2021-11-30 and 2022-04-30. ^a^ LLM, large language model. ^b^ NGASR, nurses’ global assessment of suicide risk.

As highlighted in (Fig. 4, panel A), the observer agreement analysis, few-shot prompting at temperature 0 achieved highest agreement for low risk cases (α = 0.78 [0.68, 0.88]), while zero-shot showed poorest agreement (α = 0.39 [0.24, 0.53]). Higher temperatures minimally affected few-shot performance but degraded chain-of-thought agreement from α = 0.72 [0.62, 0.83] to α = 0.67 [0.54, 0.79]. For moderate risk cases, all prompting styles demonstrated weak agreement, with chain-of-thought and few-shot at temperature 0 showing numerically higher values (α = 0.33 [0.19, 0.48]), though confidence intervals overlap with other approaches, not indicating evidence for performance differences. Agreement declined with temperature increases, most notably in chain-of-thought dropping to α = 0.20 [0.03, 0.37] at temperature 1. In high risk evaluations, zero-shot demonstrated strongest agreement (α = 0.67 [0.55, 0.80]) at temperature 0, maintaining stability across temperatures, while few-shot and chain-of-thought showed marked degradation with increased temperatures, dropping to α = 0.34 [0.15, 0.52] and α = 0.35 [0.17, 0.52] respectively. For very high risk cases, few-shot at temperature 0 achieved highest agreement (α = 0.78 [0.67, 0.89]), with all styles maintaining relatively stable performance. Zero-shot demonstrated most consistent agreement (α = 0.75 [0.64, 0.86]) across temperature settings. Lastly, sum scores revealed systematic disagreement across all configurations, with negative α values deteriorating at higher temperatures. Few-shot at temperature 0 showed least disagreement (α =  − 0.58 [− 0.65, − 0.50]), while chain-of-thought at temperature 1 demonstrated strongest disagreement (α =  − 0.90 [−0.94, − 0.86]). See also (Table 3).

### Classification performance

**Fig. 5:**
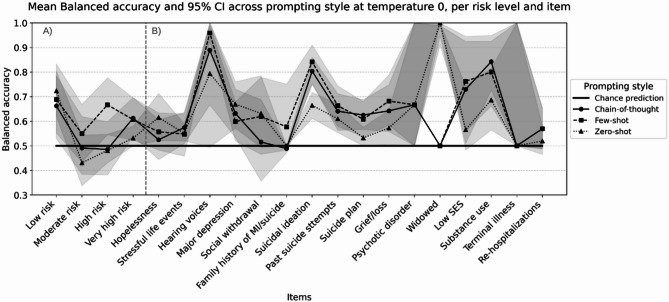
Balanced accuracy values comparing human (n = 4) and LLM^a^ ratings (N = 30) Across prompting styles at temperature 0, panel (**A**): aggregated per item, panel (**B**): aggregated per NGASR^b^ risk level (N = 2700 each), values shown as Mean and 95% Confidence Interval, assessing the presence of suicide risk factors based on n = 100 session transcripts of german youth crisis helpline users between 2021-11-30 and 2022-04-30. ^a^ LLM, large language model. ^b^ NGASR, Nurses’ Global Assessment of Suicide Risk.

The LLM framework demonstrated distinct performance patterns across risk levels as highlighted in (Fig. 5, panel A). For low risk cases, performance was consistently strong across approaches (BA: 0.71–0.72 [0.67–0.79]), with zero-shot prompting showing numerically highest sensitivity (0.93 [0.81–1.00]) while exhibiting lower specificity (0.49 [0.42–0.61]), suggesting different performance trade-offs rather than clear superiority. Few-shot prompting provided better balance with high specificity (0.92 [0.90–0.95]), maintaining stable performance across temperature settings, this being particularly valuable as it minimizes false positives, reducing unnecessary clinical interventions while maintaining screening efficiency. Meanwhile, performance declined substantially for moderate risk cases, approaching random classification. Few-shot prompting showed marginally better results (BA: 0.54 [0.49–0.59]) with balanced sensitivity (0.42 [0.26–0.55]) and specificity (0.67 [0.58–0.74]), while temperature variations had minimal impact on classification accuracy, with overlapping confidence intervals, hencenot providing support for differences in performance. Near-random classification and lowered sensitivity may raise concerns, as missing these cases could prevent early intervention. For high risk cases, few-shot prompting demonstrated superior performance (BA: 0.67 [0.55–0.74]), achieving better sensitivity (0.62 [0.50–0.73]) and specificity (0.71 [0.66–0.82]), with detailed performance metrics across all risk levels shown in (Table S1). In contrast, zero-shot’s poor sensitivity (0.05 [0.00–0.11]) poses substantial clinical risk, despite high specificity (0.93 [0.89–0.97]). Lastly, for very high risk cases, few-shot prompting showed numerically highest balanced accuracy (BA: 0.61 [0.58–0.65]), maintaining moderate sensitivity (0.31 [0.09–0.53]) and high specificity (0.92 [0.86–0.96]) though differences between prompting approaches are not providing support for differences in performance because of overlapping confidence intervals., .Chain-of-thought showed similar performance (BA: 0.60 [0.52–0.66]) but lower sensitivity (0.30 [0.17–0.43]). Zero-shot performed worst with perfect specificity (1.00 [1.00–1.00]) but negligible sensitivity (0.06 [0.00–0.10]), making it clinically unsuitable for severe risk assessment where missed cases have the highest potential consequences. Please also see (Table 4) for a detailed breakdown of all values.

**Table 4:** Balanced Accuracy, sensitivity and specificity per operational configuration across risk levels (N = 48,000 per configuration), assessing the presence of suicide risk factors based on n = 100 session transcripts of German Youth Crisis Helpline Users between 2021-11-30 and 2022-04-30.

Risk level	Temp	Metric	Chain-of-thought	Few-shot	Zero-shot
Low	0	Balanced accuracy	0.69 95% CI: [0.62–0.79]	0.71 95% CI: [0.64–0.75]	0.71 95% CI: [0.67–0.79]
		Sensitivity	0.50 95% CI: [0.20–0.66]	0.50 95% CI: [0.35–0.77]	0.93 95% CI: [0.81–1.00]
		Specificity	0.88 95% CI: [0.84–0.90]	0.92 95% CI: [0.90–0.95]	0.49 95% CI: [0.42–0.61]
	0.5	Balanced accuracy	0.71 95% CI: [0.60–0.86]	0.68 95% CI: [0.55–0.79]	0.71 95% CI: [0.67–0.77]
		Sensitivity	0.56 95% CI: [0.21–0.78]	0.43 95% CI: [0.16–0.63]	0.93 95% CI: [0.83–1.00]
		Specificity	0.85 95% CI: [0.81–0.92]	0.92 95% CI: [0.87–0.98]	0.49 95% CI: [0.41–0.56]
	1	Balanced accuracy	0.66 95% CI: [0.57–0.74]	0.69 95% CI: [0.56–0.76]	0.72 95% CI: [0.70–0.78]
		Sensitivity	0.56 95% CI: [0.37–0.84]	0.43 95% CI: [0.30–0.56]	0.93 95% CI: [0.76–1.00]
		Specificity	0.76 95% CI: [0.71–0.83]	0.95 95% CI: [0.90–0.97]	0.52 95% CI: [0.46–0.60]
Moderate	0	Balanced accuracy	0.53 95% CI: [0.41–0.67]	0.54 95% CI: [0.49–0.59]	0.44 95% CI: [0.33–0.51]
		Sensitivity	0.40 95% CI: [0.29–0.58]	0.42 95% CI: [0.26–0.55]	0.25 95% CI: [0.15–0.47]
		Specificity	0.66 95% CI: [0.59–0.69]	0.67 95% CI: [0.58–0.74]	0.63 95% CI: [0.56–0.69]
	0.5	Balanced accuracy	0.51 95% CI: [0.43–0.58]	0.56 95% CI: [0.49–0.65]	0.44 95% CI: [0.40–0.60]
		Sensitivity	0.44 95% CI: [0.36–0.54]	0.46 95% CI: [0.31–0.61]	0.25 95% CI: [0.08–0.34]
		Specificity	0.58 95% CI: [0.53–0.72]	0.66 95% CI: [0.59–0.73]	0.63 95% CI: [0.58–0.69]
	1	Balanced Accuracy	0.49 95% CI: [0.40–0.56]	0.55 95% CI: [0.48–0.57]	0.43 95% CI: [0.34–0.45]
		Sensitivity	0.32 95% CI: [0.29–0.49]	0.46 95% CI: [0.37–0.57]	0.25 95% CI: [0.12–0.47]
		Specificity	0.66 95% CI: [0.60–0.76]	0.64 95% CI: [0.56–0.70]	0.61 95% CI: [0.54–0.73]
High	0	Balanced accuracy	0.51 95% CI: [0.42–0.58]	0.59 95% CI: [0.47–0.72]	0.49 95% CI: [0.45–0.52]
		Sensitivity	0.23 95% CI: [0.09–0.33]	0.48 95% CI: [0.33–0.65]	0.05 95% CI: [0.00–0.11]
		Specificity	0.80 95% CI: [0.78–0.86]	0.70 95% CI: [0.62–0.74]	0.93 95% CI: [0.89–0.97]
	0.5	Balanced accuracy	0.47 95% CI: [0.43–0.53]	0.66 95% CI: [0.52–0.70]	0.49 95% CI: [0.44–0.50]
		Sensitivity	0.14 95% CI: [0.05–0.17]	0.57 95% CI: [0.42–0.76]	0.05 95% CI: [0.00–0.12]
		Specificity	0.80 95% CI: [0.76–0.86]	0.74 95% CI: [0.66–0.82]	0.93 95% CI: [0.88–0.97]
	1	Balanced accuracy	0.49 95% CI: [0.39–0.54]	0.67 95% CI: [0.55–0.74]	0.48 95% CI: [0.44–0.55]
		Sensitivity	0.23 95% CI: [0.05–0.27]	0.62 95% CI: [0.50–0.73]	0.05 95% CI: [0.00–0.22]
		Specificity	0.74 95% CI: [0.69–0.82]	0.71 95% CI: [0.66–0.82]	0.91 95% CI: [0.85–0.95]
Very High	0	Balanced accuracy	0.60 95% CI: [0.52–0.66]	0.61 95% CI: [0.58–0.65]	0.53 95% CI: [0.52–0.58]
		Sensitivity	0.30 95% CI: [0.17–0.43]	0.31 95% CI: [0.09–0.53]	0.06 95% CI: [0.00–0.10]
		Specificity	0.89 95% CI: [0.81–0.92]	0.92 95% CI: [0.86–0.96]	1.00 95% CI: [1.00–1.00]
	0.5	Balanced accuracy	0.58 95% CI: [0.55–0.66]	0.60 95% CI: [0.58–0.67]	0.53 95% CI: [0.52–0.58]
		Sensitivity	0.27 95% CI: [0.15–0.44]	0.28 95% CI: [0.12–0.43]	0.06 95% CI: [0.03–0.15]
		Specificity	0.89 95% CI: [0.84–0.95]	0.92 95% CI: [0.88–0.94]	1.00 95% CI: [1.00–1.00]
	1	Balanced accuracy	0.61 95% CI: [0.55–0.64]	0.61 95% CI: [0.56–0.65]	0.53 95% CI: [0.50–0.56]
		Sensitivity	0.27 95% CI: [0.15–0.37]	0.28 95% CI: [0.28–0.46]	0.06 95% CI: [0.00–0.11]
		Specificity	0.95 95% CI: [0.91–0.98]	0.93 95% CI: [0.88–0.95]	1.00 95% CI: [1.00–1.00]

Values shown as Mean with 95% Confidence Intervals. Balanced Accuracy represents the mean of sensitivity and specificity. Best values per metric within each prompting style and temperature combination are shown in bold. Temperature affects model output randomness (0 = deterministic, 1 = most random).

### Item specific analysis

Observer agreement varied across NGASR items at temperature 0, with distinct patterns for different item types (Fig. 4, Panel A). Complete reliability and agreement values for all items detailed in (Table S2). Behaviorally-anchored items showed highest agreement: hearing voices demonstrated consistently high agreement across all approaches (human α = 1.00, chain-of-thought α = 0.95 95% CI: [0.90–1.00], few-shot α = 0.91 95% CI:[0.84–0.98], zero-shot α = 0.97 95% CI: [0.93–1.01]), with overlapping confidence intervals indicating no evidence for performance differences in behaviorally-anchored items. . Items requiring clinical inference showed lower agreement: hopelessness assessment demonstrated poor agreement (human α = 0.62, chain-of-thought α = 0.24 95% CI: [0.06–0.41], few-shot α = 0.29 95% CI: [0.12–0.46], zero-shot α = 0.42 [0.26–0.58]).

Classification metrics revealed similar patterns (Fig. 5, Panel B), with comprehensive item-level performance data presented in (Table S3). Behavioral items showed strong performance: hearing voices achieved high balanced accuracy with few-shot prompting (BA = 0.97 95% CI: [0.94–0.99]). Complex clinical items performed near random: social withdrawal showed BA = 0.62 95% CI: [0.47–0.77] despite high human reliability (α = 0.92). Few-shot prompting achieved numerically highest balanced accuracy for suicide ideation (BA = 0.80 95% CI: [0.69–0.89]), though overlapping confidence intervals with chain-of-thought do not indicate differences in perfromance.

Sensitivity varied by item type and prompting style. Few-shot excelled with behavioral items (hearing voices: 1.00 95% CI: [1.00–1.00]), while zero-shot struggled with suicide assessment (suicide plan: 0.04 95% CI: [0.00–0.11]). All prompting styles maintained high specificity, particularly for observable factors (hearing voices—chain-of-thought: 0.98 95% CI: [0.94–1.00], few-shot: 0.93 95% CI: [0.88–0.98], zero-shot: 0.99 95% CI: [0.96–1.00]). Sensitivity patterns across items and risk levels are illustrated in (Fig. S1).

Regarding the tradeoff between sensitivity and specificity, Zero-shot excelled in specificity but struggled with sensitivity, particularly for suicide-related items. Few-shot achieved the most balanced trade-off, maintaining good sensitivity without sacrificing specificity. Chain-of-thought showed moderate performance in both metrics but with less extreme trade-offs. This suggests that improvements in sensitivity often came at minimal cost to specificity, particularly for few-shot prompting Specificity performance is detailed in (Fig. S2).

### Error analysis

Our error analysis revealed critical inconsistencies and logical failures in clinical reasoning, even under identical conditions. Using chain-of-thought prompting across temperatures, the model not only provided contradictory assessments but also demonstrated fundamental logical errors in clinical judgment. In one striking example, the model concluded: “while there are indications of suicidal thoughts 95% CI: […] there is no explicit expression of current suicidal ideation” despite previously noting “the patient confirms having a plan for suicide.” This represents a severe logical error, as the presence of a suicide plan necessarily implies suicidal ideation. In another case with similar input, the model imposed hallucinated diagnostic criteria: “while the patient frequently discusses their intense suicidal thoughts, they do not express any actual suicidal ideation in terms of having a plan or intent.” Yet, given similar input under identical operational conditions, it correctly identified suicidal ideation based solely on thought content: “the patient expresses suicidal ideation with an intensity of 65 out of 100[…]” These inconsistencies and logical failures suggest that despite the appearance of structured clinical reasoning through step-by-step analysis, the model lacks thorough understanding of the hierarchical and logical relationships between clinical concepts.

## Discussion

This study evaluated the performance of a LLM for standardized psychological risk assessments using the Mixtral-8x7B model under a RAG framework. We assessed the LLM’s ability to rate binary items from the Nurses’ Global Assessment of Suicide Risk (NGASR) in German crisis text line transcripts, focusing on different prompting Styles (zero-shot, few-shot, chain-of-thought) and temperature settings, which in combination we call operational configurations.

### Principal results

Our analysis revealed distinct patterns in LLM performance across reliability, observer agreement, and classification metrics. While LLMs demonstrated high internal consistency, particularly at temperature 0, this reliability did not translate to clinical validity. Zero-shot prompting achieved numerically highest internal consistency but showed poor alignment with human ratings, especially for complex clinical judgments. Few-shot prompting offered better balance, achieving numerically strongest human-AI agreement for very high risk categories, though agreement remained only moderate overall, though performance differences between approaches were often accompanied by overlapping confidence intervals, hence not supporting differences in performance.

Classification performance highlighted critical limitations in risk assessment. The framework performed best for low risk cases but approached random classification for moderate risks. Few-shot prompting at temperature 0 showed numerically higher balance for initial screening, though performance differences between approaches albeit not providing evidence for differences in performance because of overlapping confidence intervals. , While zero-shot showed concerning patterns of high specificity but negligible sensitivity for high risk cases—a limitation particularly problematic in suicide risk assessment, where not correctly identifying suicidal cases as such cases (false negatives) could have catastrophic consequences. Notably, sensitivity decreased with increasing risk levels across all prompting styles. While structured prompting improved surface-level metrics, detailed examination revealed persistent issues in clinical reasoning consistency. Given these limitations, current LLM capabilities fall short of requirements for fine-grained clinical assessment, necessitating mandatory clinical verification for moderate to high risk cases and emphasizing that LLMs should augment rather than replace clinical judgment.

Item-level analysis revealed clear performance patterns based on item characteristics. The framework performed well on behaviorally-anchored items like hearing voices but struggled with items requiring complex clinical inference such as hopelessness assessment. Few-shot prompting showed advantages for suicide-related items, though performance remained below human agreement levels. These patterns suggest that LLM effectiveness varies significantly with the type of clinical judgment required, performing best when assessing concrete, observable factors rather than interpretative clinical concepts.

### Merits and limitations

Our study offered valuable ecological validity by analyzing real clinical data from a German crisis text line, though generalizability is limited by the narrow demographic scope (female youth) and potential language model biases in youth communication patterns. We focused on female participants due to dataset composition and the need for sample homogeneity given our limited sample size due to computational constraints. Since suicide risk factors vary significantly between genders, LLMs trained on female communication patterns may not generalize to male populations. Additionally, our German-language evaluation does not indicate equivalent performance in other languages, despite Mixtral-8x7B’s multilingual pre-training^59^.

Given our limited sample size (100 cases), we prioritized sample homogeneity to ensure adequate statistical power for reliable LLM assessment. However, this design choice limits generalizability to male and gender-diverse populations. LGBTQ + youth experience suicide attempt rates 2–3 times higher than their peers, highlighting the importance of future research expanding to these high-risk populations to ensure AI tools perform equitably across all demographic groups requiring effective interventions.

An important methodological consideration is the potential relationship between transcript length and risk factor detection. Our analysis confirmed that high risk and very high risk cases were associated with longer conversations, which could partially explain improved detection rates due to greater textual exposure rather than solely higher actual risk levels. This exposure bias represents a limitation in text-based risk assessment that should be considered in future implementations.

The systematic comparison of prompting styles and temperatures revealed reliability-performance trade-offs, but excluded temporal crisis dynamics and multimodal assessment factors. Our evaluation framework included confidence intervals and multiple reliability metrics, though binary classification may oversimplify risk progression. Item-level analysis distinguished between behavioral and interpretative assessments, despite uneven base rates affecting discriminative ability measurement. Indeed, base rates significantly influenced our statistical measures, particularly balanced accuracy and regression bias-corrected Krippendorff’s α. Items with extreme base rates (e.g., terminal illness, hearing voices) showed high volatility, easily becoming “all-or-nothing” classifications where small changes in agreement patterns produced dramatic metric fluctuations. This phenomenon is mathematically expected: balanced accuracy averages sensitivity and specificity, making it sensitive to class imbalance effects even when designed to address them. Similarly, regression bias-corrected α coefficients are influenced by the distribution of positive and negative cases, with extreme base rates reducing the statistical power to detect meaningful agreement patterns.

The technical implementation featured state-of-the-art components but faced limitations in embedding quality variability and chunk size optimization. Embedding quality variability refers to inconsistent semantic representations across different text segments—essentially, some parts of conversations were converted into numerical representations that better captured their meaning than others, leading to uneven retrieval performance where relevant information might be missed if poorly represented. Chunk size optimization involved balancing context capture with computational efficiency and coherent semantic boundaries—in simpler terms, determining the optimal length of text segments to process: longer chunks provide more context but require more computational resources and may dilute important information, while shorter chunks are computationally efficient but may lose important contextual relationships between concepts. While demonstrating research feasibility, the reliance on high-performance GPUs limits practical scalability. Expert clinical ratings provided quality ground truth data, though rater diversity and expertise variations weren’t explored. The relationship between confident but incorrect LLM responses deserves deeper examination, as the nature and reason for hallucination were not the focus of this work. Overall, results reflect one specific implementation choice rather than inherent LLM capabilities, suggesting potential for alternative approaches.

Temperature 0.0 theoretically produces identical outputs, yet we observed minimal variability likely due to mixture-of-experts routing decisions sensitive to numerical precision. The near-perfect reliability (α = 1.00) confirms predominantly deterministic behavior, while majority voting maintained methodological consistency across temperatures. Also, temperature 0.0’s numerically higher performance may reflect genuine advantages of deterministic processing and a methodological limitation: higher temperatures produce variable outputs potentially requiring more than 30 repetitions for stable consensus.

Establishing ground truth in psychological assessment presents fundamental challenges. We used majority consensus from four expert raters as our criterion standard, inheriting human clinical judgment limitations. The moderate human-AI agreement may reflect an inherent ceiling effect rather than solely AI limitations. Our regression bias correction accounts for the dependency between human consensus reliability and potential human-AI agreement, acknowledging that LLM performance is mathematically constrained by human rating reliability. Where human raters showed lower consensus (moderate and high risk categories), LLMs similarly demonstrated reduced performance—aligning with known psychological risk assessment challenges rather than indicating fundamental AI failures.

An important limitation of this study relates to the NGASR scale itself, which was not originally designed for youth populations. The scale’s applicability to adolescents may be limited by developmental considerations not accounted for in its original validation. Furthermore, several NGASR items provide minimal scoring instructions, creating inherent ambiguity that challenges both human raters and LLMs. The challenges we observed in clinical assessment reliability align with findings from the original German NGASR validation study (Kozel et al. 2007), which similarly identified rater-pair agreement issues with complex clinical judgment items, particularly noting poor agreement for items like ‘social withdrawal’ that require clinical inference rather than behavioral observation. Where human raters struggled to achieve consensus (particularly for moderate and high risk categories), the LLM similarly demonstrated lower performance. This pattern is mathematically expected given that regression bias corrected Krippendorff’s α is dependent on human agreement levels, creating a ceiling effect on potential human-AI agreement. The strong performance observed in low and very high risk categories, contrasted with poorer results in moderate risk assessment, may therefore reflect inherent psychometric limitations of the scale rather than solely AI capability constraints.

### Comparison with prior work

Our study advances the emerging field of LLM applications in psychological assessment through three key contributions: implementation of state-of-the-art prompting frameworks, extension into psychological rather than purely medical assessments, and validation on real-world clinical data. While previous research has demonstrated LLMs’ potential in medical contexts, with Singhal et al.^11^ achieving notable accuracy on MedQA exam questions using Flan-PaLM, psychological applications present unique challenges requiring specialized approaches. Our investigation bridges the gap between theoretical benchmarks and practical psychological assessments by implementing sophisticated prompting frameworks in mental health contexts. Recent developments in psychological applications of LLMs have shown promising directions but remained largely experimental. Yang et al.^60^ developed the PsyCoT framework for personality trait detection, while Chen et al.^61^ focused on cognitive distortion detection through their Diagnosis of Thought (DoT) framework. These approaches demonstrated LLMs’ potential for psychological reasoning but were limited to specific domains. Our research extends these efforts by adapting structured prompting techniques to standardized suicide risk assessment, building particularly on Wu et al.’s^62^ work on chain-of-thought prompting for diagnostic reasoning. A crucial distinction of our study lies in its use of authentic clinical data. While previous work, such as Blanco-Cuaresma’s^17^ analysis of suicide risk in Reddit comments, relied on public social media data, our study utilized real crisis helpline transcripts. This represents a significant advance in ecological validity, as it evaluates LLM performance in the actual context where such systems might be deployed. This clinical dataset allowed us to assess not only technical performance but also practical applicability in authentic healthcare settings. The comprehensive evaluation of diverse prompting styles and hyperparameters on real clinical data offers unique insights into the practical challenges of implementing LLMs in mental health assessment. Our findings contribute vital understanding of both the potential and limitations of LLMs in psychological assessment, particularly in high-stakes domains like suicide risk evaluation.

Our findings both confirm and challenge previous research. While we confirm Yang et al.’s^60^ observation that LLMs can engage in psychological reasoning, our error analysis reveals more severe limitations in clinical logic than previously reported. Similarly, while we support Wu et al.’s^62^ finding that chain-of-thought prompting can improve reasoning transparency, we found it actually decreased reliability at higher temperatures—a crucial distinction for clinical applications. Unlike Blanco-Cuaresma’s^17^ promising results with social media data, our analysis of clinical transcripts showed substantially lower performance, particularly for moderate risk cases, highlighting the challenges of real-world clinical assessment versus public data analysis.

### Clinical implications

Our findings identify three promising clinical applications for LLMs in psychological assessment. First, LLMs can serve as preliminary screening tools in high-volume clinical settings, supporting initial triage decisions. Second, they can function as decision support systems, providing structured evaluations to complement clinical judgment. Third, LLMs can help standardize assessment approaches across different clinical contexts, improving multi-site consistency. Our findings suggest a tiered clinical implementation approach leveraging LLM strengths while maintaining human oversight. We propose a risk-stratified application with mandatory expert verification at critical decision points. Our error analysis revealed fundamental inconsistencies in LLM clinical reasoning, including logical contradictions within assessments. Current LLM implementations should function as decision support tools highlighting relevant information rather than independent diagnostic instruments.

Current implementations require specific conditions for optimal performance. Temperature settings and prompting styles significantly influence assessment reliability, necessitating careful calibration. LLM performance varies across clinical indicators, performing best with concrete behavioral symptoms rather than complex clinical judgments. Performance on critical risk factors, particularly suicide-related items, remains insufficient for autonomous clinical use.

Clinical deployment of LLM-based risk assessment also raises critical ethical concerns requiring careful consideration. Implementation must ensure transparent patient consent regarding AI involvement in their care, clear delineation of clinical responsibility between human clinicians and AI systems, and robust safeguards against over-reliance on automated assessments. Given the high-stakes nature of suicide risk evaluation, ethical deployment highlights the importance of human oversight at all decision points, establishing clear liability frameworks, and implementing bias monitoring to ensure equitable care across all demographic groups.

Advancing clinical viability requires enhanced prompting strategies for consistent reasoning, robust RAG mechanisms for diverse cases, and optimized parameters and validation protocols for human-AI agreement. Implementation must address ethical considerations including informed consent, data privacy, and regulatory compliance. While our study demonstrates one possible approach, alternative implementations may yield improved performance. However, any clinical applications must be developed with careful attention to both technical performance and ethical implications, particularly in high-stakes domains like suicide risk assessment.

### Future directions

Our findings indicate key priorities for advancing LLM applications in mental health assessment. Future research should prioritize validation across diverse gender identities, age groups, and cultural backgrounds to establish the broader applicability of LLM-based suicide risk assessment tools. We need mental health-specific LLMs that better capture psychological nuances, supported by open-source development for scientific replication. Current models show basic clinical reasoning capabilities but require specialized architectures and training. Future research should consider using assessment instruments with more precise operational definitions and better validated for youth populations when evaluating AI performance in psychological assessment. Moreover, robust validation frameworks are essential, as our error analysis revealed that standard metrics may mask reasoning failures. Future protocols must detect logical inconsistencies, ensure diagnostic concept hierarchies are understood, and validate criterion consistency. The temporal aspects of assessment also need attention, as current LLMs lack mechanisms to model mental state progression over time.

Practical challenges include developing privacy-preserving clinical datasets for domain adaptation and addressing cultural-linguistic variations in psychological expression. LLM decision interpretability requires investigation, particularly regarding hallucinated criteria.

### Final conclusion

This study advances our understanding of LLM applications in psychological assessment through systematic evaluation of implementation parameters and real-world clinical data. While our findings demonstrate potential for supporting specific aspects of clinical work, particularly in initial screening and standardization of assessment procedures, they also reveal fundamental challenges in clinical reasoning that current implementations have yet to overcome. The observed pattern of decreasing sensitivity with increasing risk levels poses particular concerns for high-stakes clinical applications.

Our methodological framework, emphasizing comparison against base rates and error analysis, provides valuable guidance for future evaluations of AI systems in clinical settings. The stark contrast between surface-level performance metrics and detailed reasoning analysis emphasizes the need for more sophisticated validation approaches in clinical AI research.

Looking forward, these findings suggest that advancing LLM applications in psychological assessment requires not just technical improvements, but fundamental reconsideration of how we implement and validate AI systems in clinical contexts. While current implementations are not ready for autonomous clinical application, they point toward promising directions for human-AI collaborative systems that leverage the strengths of both automated and human assessment.

## Supplementary Information

Below is the link to the electronic supplementary material.

Supplementary Material 1

## Data Availability

The datasets generated during and/or analysed during the current study are not publicly available and cannot be shared due to the highly sensitive and confidential nature of crisis helpline chat transcripts from vulnerable individuals, including minors who cannot provide consent for data sharing. These conversations frequently contain personal details and sensitive information regarding mental health and suicidal ideation. This restriction is necessary to protect participant privacy and confidentiality and to comply with ethical guidelines and data protection regulations, including the General Data Protection Regulation (GDPR). The nature of our Institutional Review Board approval and ethical framework for this research explicitly prohibits any sharing of this data beyond the approved research team. . For data-related inquiries, the first author J.T. at julia.thomas@krisenchat.de may be contacted. For other questions, please contact the corresponding author, G.M.

## Abbreviations

C-SSRS: Columbia-suicide severity rating scale
CoT: Chain-of-thought
DoT: Diagnosis-of-thought
DR-CoT: Diagnostic-reasoning-chain-of-thought
GCP: Google cloud platform
GPU: Graphics processing unit
ICC: Intraclass correlation coefficient
LLM: Large language model
NGASR: Nurses’ global assessment of suicide risk
PsyCoT: Psychological-chain-of-thought
RAG: Retrieval-augmented generation
RLHF: Reinforcement learning from human feedback
SMoE: Sparse Mixture of Experts
