# Supplementary material for: Large language model performance versus human expert ratings in automated suicide risk assessment
Source: Sci Rep. 2025 Nov 10;15:39231. doi: 10.1038/s41598-025-22402-7 (PMC12603332; doi:10.1038/s41598-025-22402-7)
Supplement: Supplementary file 1 — Supplementary Material 1 [file 41598_2025_22402_MOESM1_ESM.docx]

Multimedia Appendix: Large Language Model Performance versus Human Expert Ratings in Automated Suicide Risk Assessment

**Table S1.** Performance metrics by chain type for NGASR^a^ risk levels (N = 2,700 per condition), assessing the presence of suicide risk factors based on n = 100 session transcripts of German youth crisis helpline users between 2021-11-30 and 2022-04-30

| **Risk Level** | **Chain Type** | **Balanced Accuracy** | **F1 Score** | **Sensitivity** | **Specificity** |
| --- | --- | --- | --- | --- | --- |
| High risk | Chain-of-thought | **0.63 (0.58-0.66)** | **0.44 (0.38-0.51)** | **0.44 (0.33-0.51)** | **0.81 (0.77-0.83)** |
|  | Few-shot | 0.58 (0.52-0.62) | 0.36 (0.30-0.40) | 0.38 (0.31-0.44) | 0.78 (0.74-0.83) |
|  | Zero-shot | 0.56 (0.52-0.60) | 0.36 (0.28-0.42) | 0.40 (0.33-0.57) | 0.72 (0.68-0.77) |
| Low risk | Chain-of-thought | **0.68 (0.64-0.71)** | **0.47 (0.38-0.58)** | **0.45 (0.31-0.48)** | 0.90 (0.87-0.92) |
|  | Few-shot | 0.52 (0.46-0.54) | 0.16 (0.06-0.21) | 0.12 (0.04-0.18) | **0.92 (0.89-0.95)** |
|  | Zero-shot | 0.50 (0.49-0.53) | 0.14 (0.08-0.23) | 0.12 (0.08-0.19) | 0.89 (0.87-0.92) |
| Moderate risk | Chain-of-thought | **0.56 (0.51-0.59)** | **0.36 (0.29-0.41)** | **0.44 (0.35-0.63)** | 0.67 (0.61-0.73) |
|  | Few-shot | 0.44 (0.39-0.48) | 0.17 (0.07-0.23) | 0.17 (0.12-0.22) | 0.71 (0.66-0.75) |
|  | Zero-shot | 0.48 (0.42-0.48) | 0.20 (0.10-0.27) | 0.19 (0.10-0.29) | **0.77 (0.70-0.78)** |
| Very high risk | Chain-of-thought | **0.66 (0.63-0.72)** | **0.52 (0.48-0.57)** | **0.41 (0.37-0.51)** | **0.91 (0.87-0.93)** |
|  | Few-shot | 0.52 (0.49-0.56) | 0.30 (0.27-0.38) | 0.25 (0.18-0.30) | 0.78 (0.73-0.82) |
|  | Zero-shot | 0.53 (0.48-0.56) | 0.35 (0.29-0.39) | 0.31 (0.26-0.40) | 0.75 (0.70-0.80) |

*Note*: Mean Balanced Accuracy, Sensitivity, Specificity and F1 values with respective 95% confidence intervals attained over majority voted values per risk level. Bold values indicate highest performance within each category. ^a^ NGASR = Nurses’ Global Assessment of Suicide Risk

**Table S2.** Item-specific reliability and observer agreement values across prompting styles at temperature = 0 (N = 144,000 total ratings), assessing the presence of suicide risk factors based on n = 100 session transcripts of German youth crisis helpline users between 2021-11-30 and 2022-04-30

| **Item** | **Human Alpha** | **Corrected Alpha** | | | **LLM Alpha** | | |
| --- | --- | --- | --- | --- | --- | --- | --- |
|  |  | Chain-of-thought | Few-shot | Zero-shot | Chain-of-thought | Few-shot | Zero-shot |
| depression | 0.75 | **0.39** | 0.06 | 0.11 | 0.91 | 0.84 | **0.95** |
| grief | **0.87** | 0.62 | **0.72** | 0.67 | 0.90 | 0.94 | **0.96** |
| hopeless | 0.62 | **0.15** | -0.25 | -0.07 | 0.84 | 0.81 | **0.96** |
| hospitalized | **0.86** | 0.57 | **0.58** | 0.54 | 0.92 | 0.86 | **0.95** |
| illness | **1.00** | **0.99** | 0.91 | **0.99** | **1.00** | 0.96 | **1.00** |
| low-SES | **0.97** | **0.69** | 0.56 | 0.43 | 0.86 | 0.86 | **0.96** |
| psychosis | **1.00** | **0.99** | 0.90 | **0.99** | **1.00** | 0.96 | **1.00** |
| substance use | 0.96 | 0.85 | 0.79 | **0.90** | 0.95 | 0.95 | **1.00** |
| social_withdrawal | **0.92** | **0.11** | 0.11 | -0.52 | 0.72 | 0.89 | **0.98** |
| stressful_life_event | 0.77 | 0.30 | **0.41** | 0.40 | 0.90 | 0.91 | **1.00** |
| suicide_attempts | **0.88** | **0.66** | 0.64 | 0.64 | 0.93 | **1.00** | **1.00** |
| suicide_family_history | **0.87** | **0.93** | 0.87 | 0.81 | 0.98 | 0.97 | **1.00** |
| suicide_ideation | 0.84 | 0.60 | **0.70** | **0.70** | 0.87 | **0.96** | 0.94 |
| suicide_plan | **1.00** | 0.53 | **0.57** | **0.57** | 0.96 | 0.90 | **1.00** |
| voices | **1.00** | **0.95** | 0.86 | 0.89 | 0.97 | 0.98 | **1.00** |
| widowed | **1.00** | 0.95 | 0.92 | **1.00** | 0.98 | 0.97 | **1.00** |
| low_risk | **0.92** | **0.75** | 0.71 | 0.67 | 0.89 | 0.97 | **0.99** |
| moderate_risk | **0.66** | 0.32 | 0.33 | **0.37** | 0.81 | 0.95 | **1.00** |
| high_risk | **0.60** | **0.53** | **0.53** | 0.43 | 0.89 | 0.94 | **1.00** |
| very_high_risk | **0.76** | **0.74** | 0.52 | 0.59 | 0.90 | 0.96 | **0.98** |
| sum_score | -0.07 | -0.70 | -0.76 | -0.74 | 0.39 | 0.62 | **0.96** |

*Note*: Table presents two key metrics for each prompting style and NGASR^a^ item: (1) Regression bias corrected Krippendorff's alpha coefficients comparing human versus LLM^b^ ratings (observer agreement), and (2) Krippendorff's alpha coefficients among LLM ratings only (interrater reliability). Bold values indicate highest agreement within each category. ^a^ NGASR = Nurses’ Global Assessment of Suicide Risk ^b^ LLM = Large Language Model

**Table S3.** Performance metrics by prompting style for NGASR items (N = 2,700 per item), assessing the presence of suicide risk factors based on n = 100 session transcripts of German youth crisis helpline users between 2021-11-30 and 2022-04-30

| **Item** | **Prompting style** | **Balanced accuracy** | **F1 score** | **Sensitivity** | **Specificity** |
| --- | --- | --- | --- | --- | --- |
| Depression | Chain-of-thought | **0.61 (0.60-0.65)** | **0.69 (0.66-0.75)** | 0.79 (0.73-0.83) | **0.44 (0.40-0.51)** |
|  | Few-shot | 0.48 (0.45-0.49) | 0.63 (0.58-0.66) | 0.80 (0.73-0.82) | 0.15 (0.08-0.23) |
|  | Zero-shot | 0.54 (0.49-0.58) | 0.67 (0.64-0.70) | **0.85 (0.81-0.87)** | 0.22 (0.13-0.31) |
| Grief | Chain-of-thought | **0.69 (0.60-0.72)** | **0.54 (0.49-0.60)** | 0.50 (0.37-0.56) | 0.88 (0.86-0.90) |
|  | Few-shot | 0.61 (0.57-0.65) | 0.39 (0.36-0.43) | 0.32 (0.25-0.40) | 0.89 (0.88-0.93) |
|  | Zero-shot | 0.68 (0.66-0.74) | 0.52 (0.47-0.60) | **0.54 (0.47-0.64)** | 0.82 (0.79-0.86) |
| Hopelessness | Chain-of-thought | **0.55 (0.52-0.56)** | **0.66 (0.63-0.71)** | 0.86 (0.80-0.92) | **0.23 (0.14-0.29)** |
|  | Few-shot | 0.47 (0.43-0.52) | 0.58 (0.52-0.62) | 0.77 (0.69-0.80) | 0.17 (0.14-0.22) |
|  | Zero-shot | 0.49 (0.48-0.51) | 0.63 (0.59-0.67) | **0.88 (0.86-0.92)** | 0.10 (0.06-0.13) |
| Hospitalized | Chain-of-thought | **0.66 (0.60-0.71)** | **0.55 (0.48-0.61)** | 0.50 (0.43-0.58) | **0.82 (0.76-0.83)** |
|  | Few-shot | 0.56 (0.54-0.63) | 0.41 (0.37-0.47) | 0.38 (0.31-0.45) | 0.74 (0.72-0.80) |
|  | Zero-shot | 0.63 (0.56-0.68) | 0.51 (0.41-0.60) | **0.50 (0.45-0.54)** | 0.76 (0.70-0.82) |
| Illness | Chain-of-thought | N/A | N/A | N/A | N/A |
|  | Few-shot | 0.49 (0.47-0.53) | 0.00 | 0.00 | 0.97 (0.96-0.99) |
|  | Zero-shot | N/A | N/A | N/A | N/A |
| Low SES | Chain-of-thought | 0.76 (0.61-0.82) | **0.31 (0.21-0.43)** | 0.69 (0.57-0.90) | **0.84 (0.80-0.88)** |
|  | Few-shot | 0.61 (0.51-0.72) | 0.15 (0.11-0.19) | 0.53 (0.46-0.66) | 0.69 (0.63-0.73) |
|  | Zero-shot | **0.83 (0.80-0.88)** | 0.27 (0.19-0.31) | **1.00 (1.00-1.00)** | 0.65 (0.61-0.68) |
| Psychosis | Chain-of-thought | 0.67 (0.61-0.83) | 0.50 (0.06-0.71) | 0.33 (0.12-0.81) | **1.00 (1.00-1.00)** |
|  | Few-shot | 0.64 (0.49-0.77) | 0.22 (0.08-0.28) | 0.33 (0.19-0.94) | 0.95 (0.92-0.97) |
|  | Zero-shot | **0.83 (0.73-0.99)** | **0.80 (0.68-0.97)** | **0.67 (0.34-0.97)** | **1.00 (1.00-1.00)** |
| Substance use | Chain-of-thought | **0.86 (0.82-0.90)** | 0.75 (0.69-0.81) | 0.78 (0.69-0.86) | 0.95 (0.93-0.98) |
|  | Few-shot | 0.75 (0.68-0.82) | 0.54 (0.46-0.63) | 0.61 (0.51-0.72) | 0.89 (0.87-0.94) |
|  | Zero-shot | 0.87 (0.84-0.93) | **0.77 (0.66-0.83)** | **0.79 (0.67-0.81)** | **0.96 (0.93-0.97)** |
| Social withdrawal | Chain-of-thought | 0.56 (0.51-0.62) | 0.24 (0.18-0.26) | 0.82 (0.77-0.89) | 0.30 (0.25-0.32) |
|  | Few-shot | **0.59 (0.56-0.67)** | **0.25 (0.21-0.33)** | 0.75 (0.65-0.92) | **0.43 (0.40-0.50)** |
|  | Zero-shot | 0.51 (0.49-0.52) | 0.22 (0.18-0.27) | **0.97 (0.91-1.00)** | 0.05 (0.03-0.07) |
| Stressful life event | Chain-of-thought | 0.55 (0.53-0.60) | 0.33 (0.29-0.40) | 0.20 (0.18-0.25) | **0.90 (0.85-0.95)** |
|  | Few-shot | **0.57 (0.53-0.60)** | **0.50 (0.45-0.56)** | **0.35 (0.31-0.39)** | 0.78 (0.76-0.85) |
|  | Zero-shot | 0.54 (0.46-0.58) | 0.46 (0.43-0.50) | 0.32 (0.27-0.35) | 0.75 (0.67-0.78) |
| Suicide ideation | Chain-of-thought | 0.77 (0.74-0.79) | 0.71 (0.68-0.75) | 0.56 (0.51-0.60) | 0.98 (0.94-1.00) |
|  | Few-shot | 0.77 (0.72-0.82) | 0.80 (0.77-0.83) | **0.70 (0.65-0.73)** | 0.83 (0.73-0.91) |
|  | Zero-shot | **0.83 (0.79-0.86)** | **0.80 (0.79-0.84)** | 0.67 (0.59-0.69) | **1.00 (1.00-1.00)** |
| Family history of HI/ suicide | Chain-of-thought | 0.54 (0.50-0.59) | 0.16 (0.05-0.27) | 0.13 (0.10-0.27) | 0.95 (0.95-0.98) |
|  | Few-shot | 0.52 (0.48-0.58) | 0.10 (0.01-0.14) | 0.08 (0.00-0.16) | 0.95 (0.94-0.98) |
|  | Zero-shot | 0.54 (0.52-0.63) | 0.15 (0.04-0.16) | 0.18 (0.02-0.29) | 0.90 (0.87-0.92) |
| Suicide plan | Chain-of-thought | 0.59 (0.54-0.61) | 0.35 (0.28-0.39) | 0.22 (0.17-0.28) | 0.95 (0.92-0.97) |
|  | Few-shot | 0.63 (0.58-0.65) | 0.48 (0.44-0.54) | 0.34 (0.29-0.43) | 0.92 (0.88-0.96) |
|  | Zero-shot | 0.60 (0.59-0.64) | 0.37 (0.34-0.42) | 0.23 (0.20-0.27) | 0.97 (0.94-1.00) |
| Suicide attempts | Chain-of-thought | 0.67 (0.64-0.68) | 0.53 (0.47-0.58) | 0.37 (0.29-0.46) | 0.96 (0.94-1.00) |
|  | Few-shot | 0.65 (0.61-0.67) | 0.51 (0.49-0.54) | 0.36 (0.30-0.39) | 0.93 (0.90-0.97) |
|  | Zero-shot | 0.62 (0.60-0.64) | 0.39 (0.33-0.45) | 0.24 (0.18-0.28) | 1.00 (1.00-1.00) |
| Voices | Chain-of-thought | 0.91 (0.78-0.94) | 0.63 (0.49-0.79) | 0.86 (0.64-0.93) | 0.96 (0.95-0.97) |
|  | Few-shot | 0.97 (0.96-0.98) | 0.63 (0.51-0.73) | 1.00 (1.00-1.00) | 0.94 (0.92-0.96) |
|  | Zero-shot | 0.93 (0.87-0.96) | 0.53 (0.40-0.62) | 0.92 (0.72-1.00) | 0.93 (0.90-0.95) |
| Widowed | Chain-of-thought | N/A | N/A | N/A | N/A |
|  | Few-shot | N/A | N/A | N/A | N/A |
|  | Zero-shot | N/A | N/A | N/A | N/A |

*Note*: Mean Balanced Accuracy, Sensitivity, Specificity and F1 values with respective 95% confidence intervals attained over majority voted item wise values per item (N=2700 each) assessing the presence of suicide risk factors based on n= 100 session transcripts of German Youth Crisis Helpline Users between 2021-11-30 and 2022-04-3


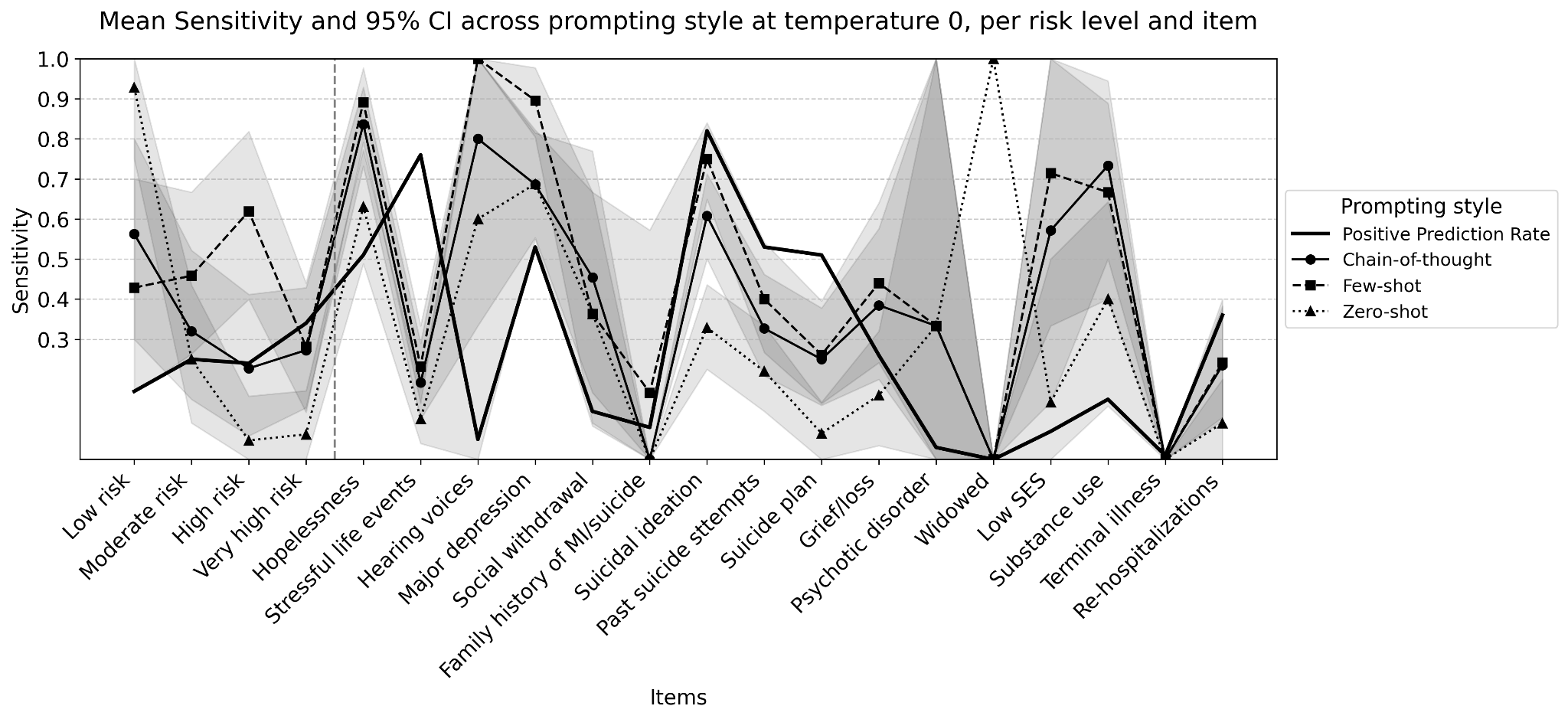


**Figure S1.** Sensitivity values comparing human (n = 4) and LLM^a^ ratings (N = 30) aggregated per item, risk level and NGASR sum score (N = 2,700 each), measured with Krippendorff's α, values shown as mean and 95% confidence interval, assessing the presence of suicide risk factors based on n = 100 session transcripts of German youth crisis helpline users between 2021-11-30 and 2022-04-30 ^a^ NGASR = Nurses’ Global Assessment of Suicide Risk ^b^ LLM = Large Language Model


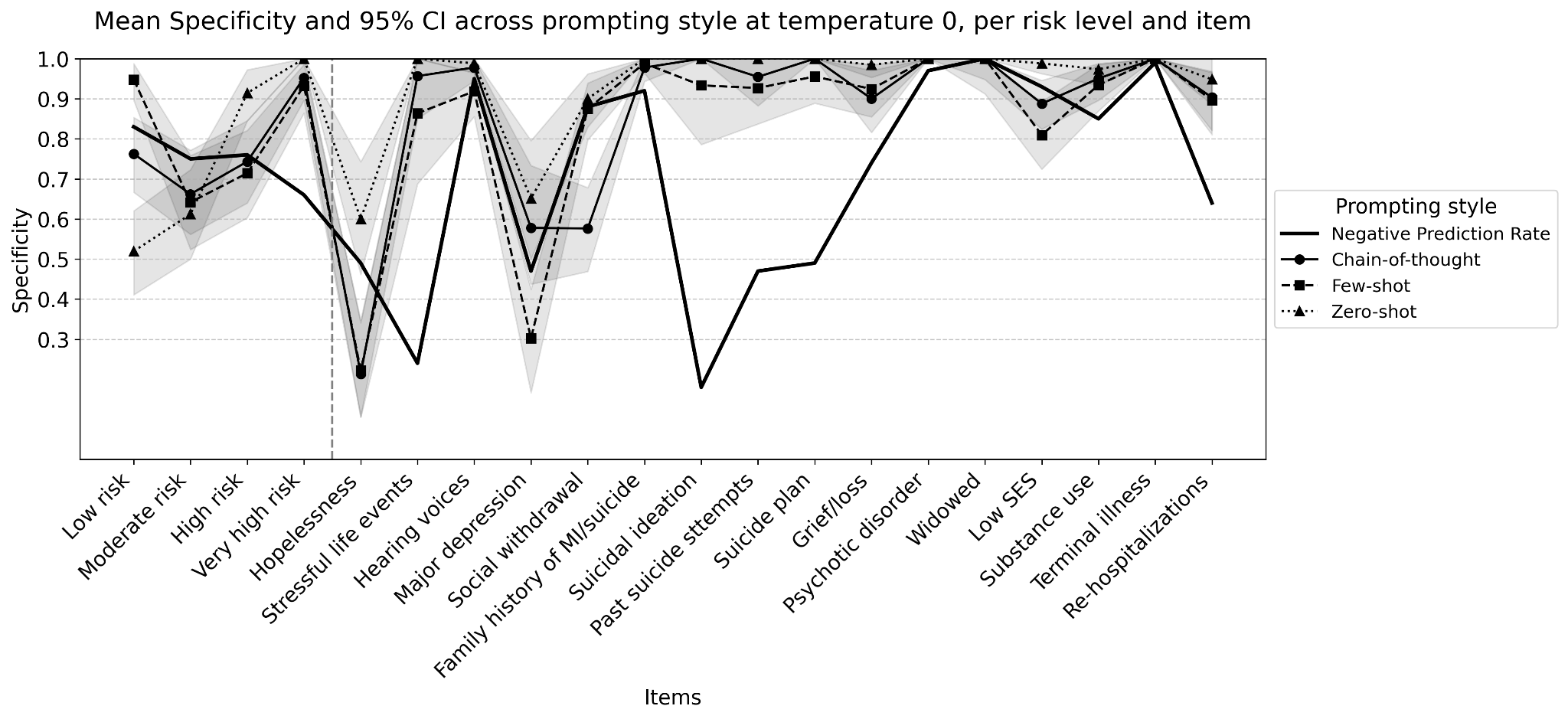


**Figure S2.** Specificity values comparing human (n = 4) and LLM^a^ ratings (N = 30) aggregated per item, risk level and NGASR^b^ sum score (N = 2,700 each), measured with Krippendorff's α, values shown as mean and 95% confidence interval, assessing the presence of suicide risk factors based on n = 100 session transcripts of German youth crisis helpline users between 2021-11-30 and 2022-04-30

^a^ NGASR = Nurses’ Global Assessment of Suicide Risk ^b^ LLM = Large Language Model


**CONSORT-AI Checklist**

**Table S4.** Consort-AI Checklist Items

## **TITLE AND ABSTRACT**

| **Section** | **Item** | **CONSORT-AI extension** | **Information from manuscript** | **Page** |
| --- | --- | --- | --- | --- |
| Title | 1a | Indicate that the intervention involves artificial intelligence/machine learning in the title and/or abstract | Title clearly states "Automated Suicide Risk Factor Monitoring" and "Using Large Language Models" | 1 |
| Abstract | 1b | Provide a structured summary including AI-specific aspects (e.g., model architecture, training approach, data characteristics, main outcome, version) | Abstract mentions "Mixtral-7x8b-Instruct" model, three temperature settings, three prompting styles (zero-shot, few-shot, chain-of-thought), and reliability metrics. | 1 |

## **INTRODUCTION**

| **Section** | **Item** | **CONSORT-AI Extension** | **Information from manuscript** | **Page** |
| --- | --- | --- | --- | --- |
| Background | 2a | Explain rationale for using an AI intervention | Introduction explains LLMs' potential in clinical psychology due to language processing capabilities, supporting information retrieval, and potential for clinical assessment | 2 |
| Background | 2b | Specify the intended use of the AI intervention | Study aims to investigate conditions for reliable psychological assessments in suicide risk evaluation by comparing LLM ratings with human expert ratings | 2-3 |

## **METHODS**

| **Section** | **Item** | **CONSORT-AI Extension** | **Information from manuscript** | **Page** |
| --- | --- | --- | --- | --- |
| Participants | 4a | Describe eligibility criteria for participants, including AI-specific considerations | Sample included female participants aged 14+ seeking help for themselves, excluding help-seeking for others | 5 |
| Interventions | 5a | Specify AI model architecture, training approach, inputs, outputs | Mixtral 8x7B model with sparse mixture of experts architecture; RAG approach with T5-based instructor-transformer embeddings | 7-8 |
| Interventions | 5b | Specify integration into the trial setting, technological infrastructure | Implementation used Python 3.8 on Google Cloud Platform Kubernetes cluster with 5-bit quantized model on 24GB L4 GPU using Ollama | 10 |
| Interventions | 5c | Describe expertise of human operators | Four expert raters from specialized suicide and self-harm counseling unit with comprehensive training on NGASR items | 6 |
| Interventions | 5d | Describe domain shift (if any) and potential for distributional shift | Not explicitly addressed, but study used authentic clinical data from the same service | - |
| Outcomes | 6a | Explain performance metrics and how they relate to the AI intervention | Used reliability analysis (Krippendorff's α), observer agreement, and classification metrics (balanced accuracy, sensitivity, specificity) | 8-10 |

## **STATISTICAL METHODS**

| **Section** | **Item** | **CONSORT-AI Extension** | **Information from manuscript** | **Page** |
| --- | --- | --- | --- | --- |
| Sample size | 7a | Detail how the sample size was determined, accounting for AI performance | Sample included 100 cases stratified by NGASR-assigned risk levels (25 cases per risk level) | 5 |
| Statistical methods | 12a | Describe methods for analyzing performance metrics and their statistical uncertainty | Used bootstrapping (1000 resamples) to compute 95% confidence intervals for α values and classification metrics | 8-9 |
| Statistical methods | 12b | Include specific methods for analyzing subgroups and interactions with the AI system | Conducted item-specific analyses using deterministic model outputs from different prompting approaches | 10 |

## **RESULTS**

| **Section** | **Item** | **CONSORT-AI Extension** | **Information from manuscript** | **Page** |
| --- | --- | --- | --- | --- |
| Participant flow | 13a | Include a flowchart showing AI-specific elements | Figure 1 presents methodological framework and data processing pipeline | 5 |
| Baseline data | 15 | Include baseline demographic and clinical characteristics | Table 1 shows demographic and clinical characteristics stratified by risk level | 14 |
| Outcomes and estimation | 17a | Report performance metrics for the AI intervention | Tables 2-4 present reliability metrics, observer agreement values, and classification performance metrics | 14-17 |

## **DISCUSSION**

| **Section** | **Item** | **CONSORT-AI Extension** | **Information from manuscript** | **Page** |
| --- | --- | --- | --- | --- |
| Limitations | 20 | Discuss limitations, including potential bias, security, safety | Discussion acknowledges limitations in risk assessment and clinical reasoning consistency | 18-20 |
| Generalizability | 21 | Discuss generalizability of the findings, considering technical factors and setting | Acknowledges limited generalizability due to narrow demographic scope and potential language model biases | 19 |
| Interpretation | 22 | Interpret findings considering benefits, harms, implementation factors | Discussion identifies three promising clinical applications while noting conditions required and limitations | 20-21 |

## **OTHER INFORMATION**

| **Section** | **Item** | **CONSORT-AI Extension** | **Information from manuscript** | **Page** |
| --- | --- | --- | --- | --- |
| Registration | 23 | Provide registration details, including version of the AI intervention | Not explicitly addressed for trial registration, but model version (Mixtral-7x8b-Instruct) is specified | 7 |
| Protocol | 24 | State where the protocol can be accessed | Study protocol received approval from Ethics Committee of IPU Berlin (approval number: 2023_08) | 11 |
| Data availability | 29 | State whether and where the AI system and validation data are available | Data availability statement indicates data cannot be shared due to sensitive nature of crisis helpline conversations | 11-12 |

# **TRIPOD-AI Checklist**

**Table S5.** Tripod-AI Checklist Items

## **TITLE & ABSTRACT**

| **Section** | **Item** | **TRIPOD-AI extension** | **Information from manuscript** | **Page** |
| --- | --- | --- | --- | --- |
| Title | 1 | Identify the study as developing or validating an AI prediction model | Title mentions "Comparative Study of AI and Human Ratings Using Large Language Models" | 1 |
| Abstract | 2 | Provide structured abstract including type of AI model, key data, target population, predictors, outcome, and results | Abstract includes model type (Mixtral-7x8b-Instruct), data source (crisis conversations), outcome (NGASR ratings), and results (reliability/accuracy metrics) | 1 |

## **INTRODUCTION**

| **Section** | **Item** | **TRIPOD-AI extension** | **Information from manuscript** | **Page** |
| --- | --- | --- | --- | --- |
| Background | 3a | Explain rationale for developing or validating the AI model | Introduction explains the need for cost-effective suicide monitoring tools and potential of LLMs for clinical assessment | 2-3 |
| Objectives | 3b | Specify the objectives, including whether the study validated existing models or developed new ones | Objectives clearly stated: to investigate conditions for reliable assessments by comparing human and LLM ratings across configurations | 3 |

## **METHODS**

| **Section** | **Item** | **TRIPOD-AI extension** | **Information from manuscript** | **Page** |
| --- | --- | --- | --- | --- |
| Source of data | 4a | Describe the study design, source of data, key dates | Study analyzed chat transcripts from German crisis text line (krisenchat) between 2021-11-30 and 2022-04-30 | 4 |
| Participants | 4b | Specify inclusion and exclusion criteria | Study included female participants aged 14+ seeking help for themselves | 5 |
| Outcome | 6a | Clearly define the outcome that is predicted | Prediction of suicide risk factors using the NGASR scale (16 items) | 5-6 |
| Outcome | 6b | Report any actions taken to blind assessment of the outcome | Four independent expert raters conducted assessments; integrity discussions conducted between rating sessions | 6 |
| Predictors | 7a | Define all predictors available at model development | All counseling transcripts were used as input for the LLM model | 5 |
| AI model development | 9a | Specify model architecture, pre-training approach, and hyperparameters | Mixtral 8x7B model with sparse mixture of experts architecture; implementation parameters described | 7-8 |
| AI model development | 9b | Describe model customization approach (fine-tuning, prompting) | Tested three prompting styles (zero-shot, few-shot, chain-of-thought) and three temperature settings | 7-8 |
| Model evaluation | 10a | Specify performance measures used | Used Krippendorff's α, observer agreement, balanced accuracy, sensitivity, specificity | 8-10 |
| Model evaluation | 10d | Justify sample size | Selected 100 cases using stratified random sampling (25 cases per risk level) | 5 |
| Risk groups | 11 | Explain and justify any grouping of predictions | Risk levels categorized as low (<4), moderate (5-8), high (9-11), and very high (≥12) per NGASR manual | 6 |

## **RESULTS**

| **Section** | **Item** | **TRIPOD-AI extension** | **Information from manuscript** | **Page** |
| --- | --- | --- | --- | --- |
| Participants | 13a | Report number of participants and events | Analysis included 100 stratified cases from a pool of 439 labeled cases | 13 |
| Model performance | 16a | Report performance measures with confidence intervals | Tables 3-4 show reliability, agreement, and classification metrics with 95% CIs | 15-17 |
| Model performance | 16b | Present both overall performance and for relevant subgroups | Results presented across risk levels, prompting styles, and temperature settings | 13-17 |
| Model updating | 17 | Report any model updates during validation | Not applicable - no model updating was performed | - |

## **DISCUSSION**

| **Section** | **Item** | **TRIPOD-AI extension** | **Information from manuscript** | **Page** |
| --- | --- | --- | --- | --- |
| Limitations | 18 | Discuss limitations including data quality, AI-specific issues | Discussion acknowledges limitations in generalizability, data scope, and technical implementation | 19 |
| Interpretation | 19a | Interpret results considering objectives, previous studies, and other models | Results compared with previous research on LLMs in psychological assessment | 20 |
| Interpretation | 19b | Discuss clinical relevance and expected benefits, harms, and costs | Discussion identifies three promising clinical applications and implementation considerations | 20-21 |
| Implications | 20 | Discuss implications for practice, AI system deployment, and future research | Future directions section outlines priorities for advancing LLM applications in mental health | 21-22 |

##

##

##

## **OTHER INFORMATION**

| **Section** | **Item** | **TRIPOD-AI extension** | **Information from manuscript** | **Page** |
| --- | --- | --- | --- | --- |
| Transparency | 21 | Provide information about model, data, code availability | Study employed open-source models with documentation of implementation steps | 10-11 |
| Funding | 22 | Provide information about funding and conflicts of interest | Funding section states "There was no funding for this study" | 23 |
